# Surface‐Confined Ultra‐Low Scale Pd Engineered Layered Co(OH)_2_ toward High‐Performance Hydrazine Electrooxidation in Alkaline Saline Water

**DOI:** 10.1002/advs.202300639

**Published:** 2023-04-29

**Authors:** Swagotom Sarker, Ji Hoon Choi, Hak Hyeon Lee, Dong Su Kim, Hyung Koun Cho

**Affiliations:** ^1^ School of Advanced Materials Science and Engineering Sungkyunkwan University (SKKU) 2066, Seobu‐ro, Jangan‐gu Suwon‐si Gyeonggi‐do 16419 Republic of Korea

**Keywords:** alkaline saline water, Co(OH)_2_, hydrazine electrooxidation, in situ redox, morphological transformation, ultra‐low scale Pd

## Abstract

Applications of abundant seawater in electrochemical energy conversion are constrained due to the sluggish oxygen evolution reaction and the corrosive chlorine oxidation reaction. Hence, it is imperative to develop an efficient anodic reaction alternative suitable for coupling with the cathodic counterpart. Due to a low thermodynamic oxidation potential, hydrazine oxidation reaction (HzOR) offers a unique pathway to overcome these challenges. Herein, spontaneously in situ reduced atomic scale Pd surface‐confined to electrochemically prepared layered Co(OH)_2_ on carbon cloth is synthesized. This study reveals the hydrazine and Pd‐dependent morphological evolution of Co(OH)_2_ and its Pd hybrids into nanoparticulate form. Unlike various layered double hydroxides, Pd integrated Co(OH)_2_ benefits from the contribution of Co(OH)_2_ as an active HzOR catalyst and the reductive support to host Pd, resulting in synergistically improved performances. Mass activities of Pd in alkaline and alkaline saline electrolyte are 11.24 and 9.83 A mg_Pd_^−1^ at 200 mV, respectively, corresponding to the highest HzOR activities among noble metals. The optimized Pd hybrid demonstrates ≈6.5 times the current density relative to Pt—C (14.91 mA cm^−2^ at 200 mV) in alkaline saline water with hydrazine. These findings would be beneficial to realize high overpotential anodic alternatives and reduce over‐dependence on freshwater for electrocatalysis.

## Graphical abstract

Hydrazine oxidation reaction (HzOR) is an alternative to high working potential anodic reactions. This demonstration with ultra‐low scale Pd surface confined to Co(OH)_2_ as a high‐performance HzOR catalyst would be beneficial to selecting reaction‐specific suitable platforms integrated with atomic catalysts in order to develop electrochemical devices utilizing abundant seawater.

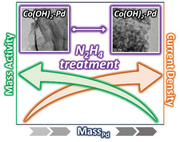

## 1 Introduction

**Figure 1:**
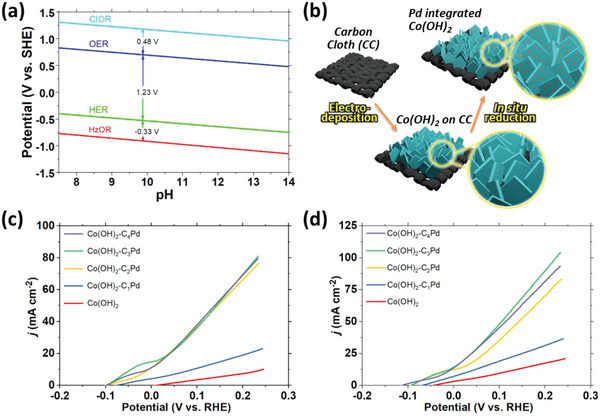
a) Schematic illustration with the Pourbaix diagram showing HzOR as a superior anodic alternative in alkaline saline water. b) Scheme for the fabrication of Pd hybrids of Co(OH)_2_ on carbon cloth. First linear sweep voltammetry (LSV) curves of Co(OH)_2_ and its Pd hybrids generated at 5 mV s^−1^ in c) 1 m KOH + 0.5 m Hz and d) 1 m KOH + 0.5 m NaCl + 0.5 m Hz.

Environmentally benign carbon‐neutral technologies such as water electrolyzers are central to combating climate change‐associated disruptions.^[^ ^1^, ^2^ ^]^ However, the efficiency of the electrochemical devices and the overall cost limit their mass‐scale adoption.^[^ ^3^, ^4^ ^]^ Overdependence on scarce freshwater, which is already overburdened due to industrial consumption, is also one of the major constraints.^[^ ^5^ ^]^ Seawater represents ≈97% of the entire water supply and could be the most promising resource as an alternative electrolyte for the electrochemical energy conversion processes to meet the global energy demand under various decarbonization initiatives.^[^ ^6^, ^7^, ^8^, ^9^ ^]^ Nonetheless, competition between oxygen evolution reaction (OER) (Equation (1)) and electrode corrosive chlorine oxidation reaction (ClOR) (Equation (2)) at high cell voltages (>1.7 V) and large current densities hinders the development of the seawater‐based electrochemical processes.^[^ ^10^, ^11^ ^]^ Kinetically sluggish OER in pure water electrolyzers also consumes ≈90% of the electricity. These limitations can be overcome by a sacrificial anodic process such as a hydrazine electrooxidation reaction (Equation (3)), which requires an exceptionally low overpotential (−0.33 V vs reversible hydrogen electrode (RHE)). Correlation among these anodic processes for the alkaline condition is illustrated via the Pourbaix diagram^[^ ^6^, ^10^, ^12^, ^13^ ^]^ presented in **Figure** **1**a. Broader applications of HzOR particularly in the field of energy conversion involve coupling it with a counter‐cathodic reaction such as hydrogen evolution reaction (HER),^[^ ^10^, ^14^, ^15^, ^16^, ^17^, ^18^ ^]^ oxygen reduction reaction (ORR),^[^ ^19^, ^20^ ^]^ and hydrogen peroxide reduction reaction.^[^ ^21^ ^]^ On a separate note, hydrazine, which is highly toxic to human health, is also used as the deoxidant in the feed water of power plants, the fuel in the space industry, and the raw materials for various industrial processes. Therefore, HzOR also paves the efficient and cost‐effective way for its eventual electrocatalytic neutralization aimed for a safer environment.

(1) $$\mathrm{OER}:4{\mathrm{OH}}^{-}\to O_{2}+2H_{2}O+4e^{-},\left(1.23\;V\;\mathrm{vs}\;\mathrm{RHE}\right)$$

(2) $$\mathrm{ClOR}:{\mathrm{Cl}}^{-}+2{\mathrm{OH}}^{-}\to {\mathrm{ClO}}^{-}+H_{2}O+2e^{-},\left(1.71\;V\;\mathrm{vs}\;\mathrm{RHE}\right)$$

(3) $$\begin{array}{c} \mathrm{HzOR}:N_{2}H_{4}+4{\mathrm{OH}}^{-}\to N_{2}+4H_{2}O+4e^{-},\left(-0.33\;V\;\mathrm{vs}\;\mathrm{RHE}\right) \end{array}$$

Literature reports indicate that the metallic phase of the transition metals (Ni, Co, and Cu) and their derivatives such as nitrides, phosphides, carbides, and chalcogenides often in the form of heterostructure interfaces and/or composites is one of the critical criteria in the HzOR catalyst design.^[^ ^13^, ^15^, ^16^, ^19^, ^22^ ^]^ P, W co‐doped Co_3_N,^[^ ^23^ ^]^ Mo‐Ni_3_N/Ni/NF,^[^ ^15^ ^]^ Ni_2_P/Zn‐Ni‐P,^[^ ^24^ ^]^ Ni_3_N‐Co_3_N,^[^ ^25^ ^]^ Co arrays on Cu foam,^[^ ^19^ ^]^ Ni–Co alloy,^[^ ^26^ ^]^ NiCo/MoNi_4_,^[^ ^22^ ^]^ Co*_x_* P@Co_3_O_4_,^[^ ^27^ ^]^ CC@CoNC,^[^ ^28^ ^]^ NiCo@C/MXene,^[^ ^10^ ^]^ CoNi‐alloy@CoNi‐sulfide^[^ ^29^ ^]^ core–shell nanoarrays, Co_NS_@Cu_PD_,^[^ ^30^ ^]^ and (Ni,Co)_0.85_Se anchored on rGO^[^ ^31^ ^]^ have been shown suitable for HzOR with varied degrees of activities. These non‐noble metal catalysts are normally fabricated via phase transformation of corresponding oxides or hydroxides by utilizing a post‐synthetic modification technique such as gas treatment at an elevated temperature or plasma treatment since composition‐tuned (layered) oxides and hydroxides have predominantly been shown poor toward HzOR,^[^ ^16^, ^29^ ^]^ unlike HER and OER.^[^ ^32^, ^33^ ^]^ Among the noble metals, derivatives of Rh and Ru such as Rh/RhO*_x_* nanosheets,^[^ ^34^ ^]^ Rh_2_S_3_/N‐Doped carbon hybrids,^[^ ^35^ ^]^ Rh_2_P/Rh@NPG,^[^ ^17^ ^]^ Ru‐Cu_2_O/CF,^[^ ^36^ ^]^ and RuP_2_^[^ ^37^ ^]^ exhibit high HzOR current densities at low working potentials. However, the aforementioned catalysts are primarily nanoparticulate. Among the few reports on nanoparticulate Pd catalyzed HzOR,^[^ ^38^, ^39^, ^40^, ^41^, ^42^ ^]^ recent work on Pd with NiFe LDH support highlights the size sensitivity of Pd toward HzOR,^[^ ^38^ ^]^ where Pd nanocluster coupled NiFe LDH outperformed with a current density of 4.3 A mg_Pd_^−1^ at 0.35 V vs RHE, which was 36 and 7 times relative to its Pd single atom and nanoparticulate counterparts, respectively. Recently, a new class of emerging catalysts, which predominantly encompasses Pt,^[^ ^43^, ^44^, ^45^, ^46^ ^]^ Pd,^[^ ^47^ ^]^ Rh,^[^ ^48^, ^49^ ^]^ Ru,^[^ ^50^ ^]^ and Ir^[^ ^51^, ^52^ ^]^ at ultra‐low concentration or in the form of single atoms obtained by ion exchange, electrodeposition, precursor concentration control, atomic layer deposition, and other methods, have demonstrated exceptional performance in OER,^[^ ^53^, ^54^ ^]^ ORR,^[^ ^46^ ^]^ and HER.^[^ ^54^ ^]^ Surprisingly, the field atomic catalyst‐enhanced HzOR has yet to receive full attention.^[^ ^55^, ^56^, ^57^ ^]^ High mass activity in addition to the geometric area normalized performances is pivotal to the atomic/ultra‐low scale catalysts involving precious metals to ascertain their cost‐effectiveness. However, achieving surface‐limited distribution of metallic atomic catalysts is the foremost challenge. While each method has its own merits and disadvantages, the in situ redox process^[^ ^58^ ^]^ is a mechanistically unique post‐synthetic technique to enable a spontaneous transformation of a relatively less active catalyst or produce a fine‐tuned surface site electrocatalyst at room‐temperature. The surface‐confined presence of the desired catalysts would be beneficial to the electron transport during electrocatalysis resulting in a higher mass activity. Overall, there exists a lack of research on the understanding of the innate activity of the layered hydroxides toward HzOR in addition to their post‐synthetic modification via incorporation of atomic scale noble metals for further enhanced electrochemical performances. We notice that a considerable number of HzOR catalysts involve cobalt;^[^ ^19^, ^22^, ^25^, ^27^, ^29^, ^59^ ^]^ whereas, Co(OH)_2_ is partially active toward HzOR.^[^ ^19^ ^]^ On the other hand, seawater contains nearly 0.5 m Na^+^ and Cl^−^ apart from significant amount of Ca^2+^, Mg^2+^, microorganisms, and other impurities.^[^ ^7^, ^60^ ^]^ Presence of Ca^2+^, Mg^2+^, and impurities in a seawater electrolyte can negatively contribute to the electrocatalytic performances. At higher pH, Ca^2+^ and Mg^2+^ precipitate as hydroxides and the purified solution resembles as the alkaline saline electrolyte containing 0.5 m NaCl.^[^ ^61^, ^62^, ^63^, ^64^ ^]^ Based on these circumstances, we hypothesize that the development of a comparative analysis between Co(OH)_2_ and morphologically analogous CoFeLDH in addition to the redox process aided surface integration of Pd at ultra‐low scale would be beneficial to realize the larger scope of the selection of the catalysts toward achieving synergistically improved HzOR in both alkaline and simulated alkaline seawater.

Herein, we report synergistically enhanced HzOR activities in both alkaline and alkaline saline electrolytes realized from the hybrids of ultra‐low scale Pd surface confined to Co(OH)_2_, which were fabricated via an in situ redox process aided integration of Pd into layered Co(OH)_2_ electrodeposited on carbon cloth (CC). First, to select a proper platform to initiate in situ reduction of Pd and its optimization toward synergistic HzOR, the roles of Fe were explored from a comparative analysis of layered Co(OH)_2_ and morphologically analogous CoFeLDH. We demonstrate that i) the presence of Fe in the hydroxides leads to the suppression of HzOR activities of both Co(OH)_2_ and Pd and ii), for the first time, layered Co(OH)_2_ and Pd hybrids morphologically evolve in presence of hydrazine. The resulting surface‐limited Pd hybrids of Co(OH)_2_ exhibit an HzOR mass activity of 11.24 and 9.83 A mg_Pd_^−1^ at 200 mV in alkaline and alkaline saline water, respectively; these are one of the most outstanding values at such low working potential reported for all noble metal‐based electrocatalysts in the respective electrolyte. Overall, synergistic enhancement toward the HzOR is reflected from the lower onset potential, the higher current densities, and the smaller Tafel slopes. The optimized Pd integrated Co(OH)_2_ requires only 200 mV of working potential to reach 96.67 mA cm^−2^ in the simulated alkaline seawater with hydrazine; whereas, commercial Pt—C exhibits only 14.91 mA cm^−2^. Structure‐activity relationship shows preferential electrocatalytic HzOR at low hydrazine concentration on metallic Pd in Co(OH)_2_‐C*_i_* Pd. Overall, apart from the demonstration of the electrocatalytic process independent morphological evolution of Co(OH)_2_ and its Pd hybrids in presence of hydrazine and achievement of superior electrocatalytic HzOR performances, this study clearly illustrates the significance of the consideration of a proper matrix and the development of the highly efficient surface limited ultra‐low scale catalysts utilizing a spontaneous in situ redox technique as part of post‐synthetic modification to harness benefits from the vast seawater.

## 2 Results and Discussion

### 2.1 Catalyst Preparation and Physical Characterization

In this work, layered hydroxides of Co and CoFe were grown on the CC under a constant charge of −1.4C at −1 V versus Ag/AgCl. in situ reduction of Pd was initiated and its content in response to the HzOR performance was optimized for its hybrids with Co(OH)_2_. The synthesis of layered Co(OH)_2_ on CC and in situ reduced Pd integration is illustrated in Figure 1b. N_2_H_4_ is known as a reducing agent and may contribute to the creation of oxygen vacancies as well as morphological development.^[^ ^65^, ^66^, ^67^ ^]^ Therefore, all as‐prepared samples were conditioned to the HzOR treatment via one linear sweep voltammetry (LSV) executed from −1.10 to −0.70 V versus Hg/HgO at 5 mV s^−1^ (discussed further in section 2.2.1 and 2.2.2) to ascertain the probable surface and morphological transformation arising from the presence of N_2_H_4_ and obtain stabilized HzOR performances in 1 m KOH and 1 m KOH + 0.5 m NaCl solutions with varied concentration of hydrazine. Pd integrated samples of Co(OH)_2_ were designated by Co(OH)_2_‐C*_i_* Pd. Here, *i* refers to various Pd exchanged samples: Co(OH)_2_‐C_1_Pd, Co(OH)_2_‐C_2_Pd, Co(OH)_2_‐C_3_Pd, and Co(OH)_2_‐C_4_Pd, where C_1_, C_2_, C_3_, and C_4_ stand for 0.01, 0.05, 0.10, and 0.20 mm 4 mL Pd solution, respectively. The first LSV scans of Co(OH)_2_ and Co(OH)_2_‐C*_i_* Pd are presented in Figure 1c,d corresponding to the HzOR treatment in 1 m KOH + 0.5 m Hz and 1 m KOH + 0.5 m NaCl + 0.5 Hz, respectively. Respective first LSVs of CoFeLDH and CoFeLDH‐C_3_Pd are shown in Figures S1a and S1b, Supporting Information. The geometric surface area normalized Co(OH)_2_‐C_3_Pd exhibits the optimum performance toward HzOR. Therefore, we opted to focus more on Co(OH)_2_‐C_3_Pd for different characterizations. After completion of the first polarization, each sample was washed with ethanol and air‐dried as part of initiating broader physical and electrocatalytic characterization.

**Figure 2:**
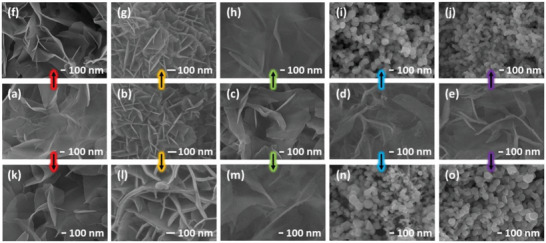
SEM images of as‐prepared a) Co(OH)_2_, b) CoFeLDH, c) Co(OH)_2_‐C_1_Pd, d) Co(OH)_2_‐C_2_Pd, and e) Co(OH)_2_‐C_3_Pd grown on carbon cloth as shown in the second row. HzOR treated sample images presented in f–j) the first row [HzOR treatment condition: 1 LSV executed at 5 mV s^−1^ in 1 m KOH + 0.5 m Hz] and k–o) the 3^rd^ row [HzOR treatment condition: 1 LSV executed at 5 mV s^−1^ in 1 m KOH + 0.5 m NaCl + 0.5 m Hz]. Arrow (⟶) used to correlate the images of the as‐prepared samples with corresponding HzOR treated samples.

The morphology of Co(OH)_2_, CoFeLDH, and their Pd hybrids obtained under different conditions was observed with field‐emission scanning electron microscopy (SEM). SEM images of pristine Co(OH)_2_ (**Figure** **2**a and Figure S2, Supporting Information) show randomly stacked 3D macroporous cross‐linked layered nanosheets on CC. Low and high‐magnification SEM images of Co(OH)_2_ reveal complete coverage of CC with interconnected nanosheets. Pristine CoFeLDH also exhibits compact structures made of LDH nanosheets (Figure 2b) although an individual layer of Co(OH)_2_ appears relatively thinner than that of CoFeLDH. As‐prepared Co(OH)_2_‐C*_i_* Pd (Figure 2c–e and Figure S3a, Supporting Information) and CoFeLDH‐C_3_Pd (Figure S4, Supporting Information) hybrids also maintain the original structure of Co(OH)_2_ and CoFeLDH. After one linear sweep polarization in 1 m KOH + 0.5 m Hz, no noticeable changes in the morphology of Co(OH)_2_ (Figure 2f) and CoFeLDH (Figure 2g) are observed. Co(OH)_2_‐C_1_Pd, which is expected to contain negligible amount of Pd, also demonstrates a feature similar to its as‐prepared counterpart in the form of layered structure (Figure 2h). Corresponding SEM images of HzOR‐treated Co(OH)_2_‐C_2_Pd and Co(OH)_2_‐C_3_Pd are presented in Figure 2i,j; whereas, Figure S3b, Supporting Information, features the SEM image of Co(OH)_2_‐C_4_Pd treated with HzOR. However, we observe that Co(OH)_2_‐C_2_Pd, Co(OH)_2_‐C_3_Pd, and Co(OH)_2_‐C_4_Pd hybrids, which were obtained via in situ redox method for Pd content ≥0.01 mm in 4 mL, exhibit a surprising morphological transformation. Layered structures of these hybrids morph into nanoparticulate forms of irregular geometry while the morphology of HzOR‐treated Co(OH)_2_ and Co(OH)_2_‐C_1_Pd remains unchanged relative to their pristine structures. HzOR treatment results in the formation of both agglomeration and nano‐islands of these layered hybrids attached to CC. At this initial stage, we can assume that morphological transformation of Pd hybrids of Co(OH)_2_ is linked to Pd and electrocatalytic hydrazine oxidation. We also obtained the SEM images of Co(OH)_2_ (Figure 2k), CoFeLDH (Figure 2l), Co(OH)_2_‐C*_i_* Pd (Figure 2m–o and Figure S3c, Supporting Information) samples that were subjected to one linear polarization in 1 m KOH + 0.5 m NaCl + 0.5 m Hz. They are identical to their alkaline HzOR‐treated corresponding counterparts. Therefore, we can conclude that the morphological transformation of the Pd hybrids of Co(OH)_2_ is independent of salinity in the electrolyte. Although CoFeLDH‐C_3_Pd and Co(OH)_2_‐C_3_Pd were prepared via in situ redox process with the same precursor concentration of Pd, CoFeLDH‐C_3_Pd unlike Co(OH)_2_‐C_3_Pd does not alter its layered morphology into nanoparticulate form after HzOR treatment (Figure S5a,b, Supporting Information). We infer that such structural preservation of CoFeLDH‐C_3_Pd yet anomalous when compared to Co(OH)_2_‐C_3_Pd after HzOR treatment could be due to Fe presence in its structure. Naturally, these observations made us curious to question whether the morphological transformation is a) dependent on i) hydrazine concentration and ii) Pd content in the hybrid or b) independent of HzOR but solely dependent on the presence of hydrazine in the solution. Due to the similar feature of the SEM images of the HzOR‐treated samples prepared in alkaline and alkaline saline electrolyte with hydrazine, we primarily focus on their associated changes arising from 1 m KOH with hydrazine.

**Figure 3:**
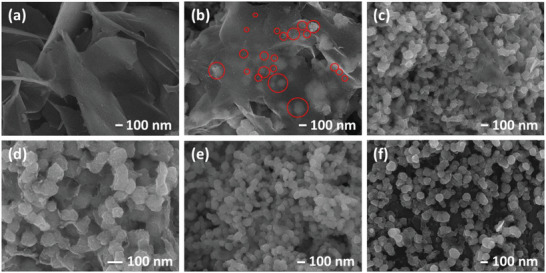
SEM images of Co(OH)_2_‐C_3_Pd after first LSV executed at 5 mV s^−1^ toward HzOR in 1 m KOH with a) 0 m, b) 0.05 m, c) 0.1 m, d) 0.2 m, and e) 0.5 m (also shown in Figure 2j) Hz. f) SEM image of as‐prepared Co(OH)_2_‐C_3_Pd after exposure for 80 s in 1 m KOH + 0.5 m Hz.

Since HzOR is an anodic process, Co(OH)_2_ and Co(OH)_2_‐C_3_Pd were also subjected to linear polarization toward OER (Figure S6, Supporting Information) from 1.045 to 1.745 V at 5 mV s^−1^ in 1 m KOH to ascertain whether the transformation of the layered structures is associated with hydrazine. Respective SEM images of OER‐treated Co(OH)_2_ and Co(OH)_2_‐C_3_Pd are presented in Figures S7a and S7b, Supporting Information, which show no noticeable change in the morphologies compared to the as‐prepared samples. These results indicate that prior observations on morphological change in both alkaline and alkaline saline electrolytes with 0.5 m Hz may be dependent on HzOR. However, we need to keep in mind that the potential windows of HzOR and OER treatment are different. To evaluate the hydrazine concentration‐dependent structural reconstruction, as‐prepared Co(OH)_2_‐C_3_Pd samples were also treated within the same potential window of HzOR with electrolytes containing 0, 0.05, 0.1, and 0.2 m Hz in 1 m KOH (Figure S8, Supporting Information). SEM images of Co(OH)_2_‐C_3_Pd were obtained (**Figure** **3**a–d and Figure S9a–d, Supporting Information) and compared to the as‐prepared (Figure 2e) and 0.5 m Hz treated counterparts (Figure 2j and Figure S9e, Supporting Information). Co(OH)_2_‐C_3_Pd sample, which was electrochemically treated in 1 m KOH + 0 m Hz, preserves its original layered structure (Figure 3a and Figure S9a, Supporting Information). However, as the concentration of hydrazine is continuously increased, a gradual morphological transformation of Co(OH)_2_‐C_3_Pd becomes visible. A single linear sweep polarization in 1 m KOH + 0.05 m Hz of the as‐prepared Co(OH)_2_‐C_3_Pd sample leads to the damage of the parent nanosheet structures and an eventual co‐existence of nanosheets connected/disconnected with the CC and nanoparticles. A closer look at the image reveals the appearance of smaller nano‐islands (shown in red circles) wrapped with or on the surface of Co(OH)_2_ nanosheets (Figure 3b and Figure S9b, Supporting Information). HzOR treatment at 1 m KOH + 0.1 m Hz results in the further disappearance of the nanosheets and the nanoparticles are mostly covered with rough edges (Figure 3c and Figure S9c, Supporting Information). The remnants of the nanosheets diminish further and nano‐particles also evolve by losing their apparent roughness (Figure 3d and Figure S9d, Supporting Information) as a result of electrochemical treatment of Co(OH)_2_‐C_3_Pd in 1 m KOH + 0.2 m Hz. This progression of transformation continues and leads to the formation of nanosheet‐free interconnected nanoparticles from its parent 2D cross‐linked layered structures (Figure 3e and Figure S9e, Supporting Information). Certainly, this phenomenon of morphological translation of as‐prepared Co(OH)_2_‐C_3_Pd is dependent on the hydrazine concentration during HzOR. Nonetheless, one could still question whether this transformation, at all, requires the HzOR treatment. Thus, we also exposed the as‐prepared Co(OH)_2_‐C_3_Pd in 1 m KOH + 0.5 m Hz for ≈80 s, which is equivalent to the duration of one LSV from −1.10 to −0.70 V at 5 mV s^−1^. The purpose of this exposure in 1 m KOH + 0.5 m Hz is to determine whether morphological transformation is independent of HzOR. In this case, Co(OH)_2_‐C_3_Pd was allowed to only pure chemical treatment without HzOR. SEM images of alkaline hydrazine solution treated Co(OH)_2_‐C_3_Pd are presented in Figure 3f and Figure S9f, Supporting Information, and morphological transformation of Co(OH)_2_‐C_3_Pd is analogous to those in SEM images presented in (Figure 3e and Figure S9e, Supporting Information). Therefore, we can state from our systematic observations that the HzOR associated with morphological evolution of Co(OH)_2_‐C_2_Pd, Co(OH)_2_‐C_3_Pd, and Co(OH)_2_‐C_4_Pd is independent of electrocatalytic HzOR but a chemical transformation of Co(OH)_2_ dependent on hydrazine concentration and Pd content. As‐prepared Co(OH)_2_ was also exposed to the alkaline hydrazine solution for ≈80 s. Both electrochemically (alkaline and alkaline saline electrolytes with 0.5 m Hz) and chemically treated Co(OH)_2_ exhibited equivalent features (Figure 2f,k and Figure S10a,b, Supporting Information). However, longer exposure to alkaline hydrazine solution (chemical treatment) results in the gradual evolution of Co(OH)_2_: from interconnected 2D nanosheets (Figure S11a, Supporting Information, exposure duration: 0 s) to the apparent preservation of nanosheets (Figure S11b, Supporting Information, exposure duration: 80 s), the formation of smaller fissures on the nanosheet surface (Figure S11c, Supporting Information, exposure duration: 480 s), and finally, the transformation of the nanosheets into smaller structures of non‐uniform thickness and irregular geometry (Figure S11d, Supporting Information, exposure duration: 1600 s). Electrochemical HzOR treatment of Co(OH)_2_ in 1 m KOH + 0.5 m Hz results in comparable structural reconstruction, as shown in Figures S12b (1 LSV; exposure duration: 80 s), S12c (6 LSVs; exposure duration: 480 s), and S12d (20 LSVs; exposure duration: 1600 s), Supporting Information. In addition, Co(OH)_2_‐C_1_Pd (Figure S13a,b, Supporting Information) shows the existence of both edged nanoparticles and nanosheets after three LSVs in 1 m KOH + 0.5 m Hz. Therefore, we speculate that the transformation rate of the layered Co(OH)_2_ may be more specific to the location of Pd on its surface. Furthermore, CoFeLDH‐C_3_Pd also shows the formation of nanoparticles of irregular geometry from its layered structure after 20 LSVs in 1 m KOH + 0.5 m Hz (Figure S14a,b, Supporting Information) yet different in shape from that of Co(OH)_2_‐C_3_Pd (Figure 3e). However, CoFeLDH effectively remains unchanged (Figure S15a,b, Supporting Information; 20 LSVs; exposure duration: 1600 s). These observations lead us to reasonably conclude that the hydrazine‐induced structural reconstruction is i) slower in the absence of Pd, ii) dependent on the elemental composition of the hydroxide and Pd, and iii) independent of electrocatalytic HzOR. However, the shapes and sizes of the morphed nanosheets appear to be dependent on Pd. Therefore, we suggest that layered hydroxides and their derivatives should be treated in a hydrazine‐containing electrolyte before the measurement of the overall electrocatalytic HzOR. Energy‐dispersive X‐ray (EDX) analyses of Co(OH)_2_‐C_3_Pd (Figure S16a,b, Supporting Information) and CoFeLDH‐C_3_Pd (Figure S17a,b, Supporting Information) show the presence of relevant elements in their patterns with Pd contents at 0.4 (Table S1, Supporting Information) and 0.44 atomic % (Table S2, Supporting Information), respectively. Thus, Pd content appears comparable in both samples and EDX‐elemental mappings of these samples portray a distinct and uniform distribution of Pd on the hydroxide surfaces confirming the successful fabrication of the hybrids via in situ redox method (Figures S18 and S19, Supporting Information).

**Figure 4:**
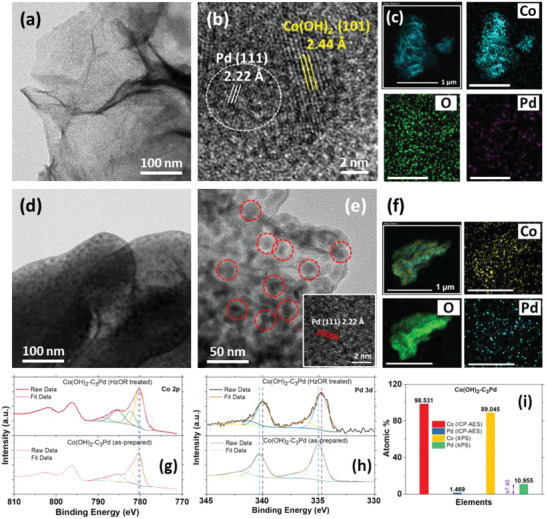
a,b) (HR)TEM images with corresponding c) HRTEM‐EDS elemental mappings of as‐prepared Co(OH)_2_‐C_3_Pd. d,e) (HR)TEM images with an inset in (e) and corresponding HRTEM‐EDS elemental mapping in (f) of Co(OH)_2_‐C_3_Pd after 1 LSV executed from −1.1 to −0.70 V versus Hg/HgO at 5 mV s^−1^ in 1 m KOH + 0.5 m Hz. g) Co 2p and h) Pd 3d XPS spectra of Co(OH)_2_‐C_3_Pd. i) Evaluation of Co and Pd composition in as‐prepared Co(OH)_2_‐C_3_Pd via XPS and inductively coupled plasma‐optical emission spectroscopy (ICP‐OES).

The X‐ray diffractometer (XRD) patterns of as‐prepared Co(OH)_2_‐C_3_Pd and HzOR‐treated Co(OH)_2_‐C_3_Pd showed no clear peaks of Co(OH)_2_ and Pd (Figure S20, Supporting Information). It could be due to the very thin layered film of Co(OH)_2_ on CC and the low concentration of Pd. The transmission electron microscopic (TEM) image of as‐prepared Co(OH)_2_‐C_3_Pd demonstrates the feature of ultra‐thin nanosheets without any presence of large clusters or agglomeration of Pd as shown in **Figure** **4**a. A high‐resolution TEM (HRTEM) image (Figure S21, Supporting Information) shows that atomic scale Pd (red circles) is evenly distributed over the layered Co(OH)_2_. The HRTEM image in Figure 4b shows the interplanar distances of Pd (2.22 Å) and Co(OH)_2_ (2.44 Å).^[^ ^68^, ^69^, ^70^ ^]^ The selected area diffraction (SAD) pattern was obtained from TEM analysis of as‐prepared Co(OH)_2_‐C_3_Pd and is presented in Figure S22, Supporting Information. Co(OH)_2_ with the (100) and (101) crystal orientation related to 2.75 and 2.44 Å, respectively (corresponding JCPDS No. 46–0605), and Co_3_O_4_ corresponding to 1.43 Å were identified (JCPDS No. 42–1467). The presence of Co_3_O_4_ could be due to the oxidation of the Co(OH)_2_ monolayer by the high electron beam energy of the TEM, which occurs in a very short time. However, we have identified clear Pd (111) crystallinity, which has an interplanar distance of 2.22 Å. TEM elemental mappings of as‐prepared Co(OH)_2_‐C_3_Pd are presented in Figure 4c. It shows the coexistence of Co, Pd, and O in the catalyst in addition to a uniform distribution of Pd on the Co(OH)_2_ matrix. In particular, it can be confirmed that the Pd is uniformly distributed in a small amount. To further investigate the local structures, we also conducted the (HR)TEM analysis of the Co(OH)_2_‐C_3_Pd sample, obtained via the HzOR treatment of one sweep of LSV in 1 m KOH + 0.5 m Hz. In contrast to Figure 4a, the TEM image of HzOR‐treated Co(OH)_2_‐C_3_Pd shows the absence of a thin layered structure and the presence of Pd with relatively sharper and larger black spots (further highlighted in Figure 4e with an inset of the interplanar distance of Pd). Therefore, an atomic scale distribution of Pd in as‐prepared Co(OH)_2_‐C_3_Pd is transformed to a larger distribution of dimension in HzOR‐treated Co(OH)_2_‐C_3_Pd during the morphological evolution. It is probable that neighboring atomic Pd in as‐prepared Co(OH)_2_‐C_3_Pd gradually contributes a larger size distribution of Pd as the structural transformation progresses during the HzOR treatment. The corresponding SAD pattern is also presented in Figure S23, Supporting Information, showing the presence of Co(OH)_2_ and Pd. TEM elemental mappings of the HzOR‐treated Co(OH)_2_‐C_3_Pd also confirm the coexistence and the distribution of Co, O, and Pd in the transformed morphology as well as a relatively larger size of Pd (Figure 4f).

X‐ray photoelectron spectroscopy (XPS) analysis was carried out to inspect the elemental composition and the valence states in Co(OH)_2_‐C_3_Pd and its electrochemically treated counterpart prepared via one sweep of linear polarization in 1 m KOH + 0.5 m Hz. The spectra (Figure S24a,b, Supporting Information) show the coexistence of Co, Pd, and O, and the deconvoluted XPS spectra of Co and Pd are presented in Figures 4g,h, respectively. The Co 2p_3/2_ XPS peak was deconvoluted and identified at 780.35, 782.36, 785.40, and 788.75 eV indicating the existence of Co^2+^ in the as‐prepared Co(OH)_2_‐C_3_Pd sample.^[^ ^67^, ^68^, ^71^ ^]^ After the HzOR treatment of Co(OH)_2_‐C_3_Pd, the corresponding deconvoluted peaks are located at 780.09 and 782.24 eV along with satellite peaks at 785.34 and 788.44 eV. The slight shift to the lower binding energy may be attributed to the hydrazine‐induced reduction of the oxidation state of cobalt (Co^2−^ *^*δ*^*).^[^ ^19^, ^71^, ^72^ ^]^ The respective deconvoluted peaks at 335.01 and 340.27 eV can be assigned to the Pd 3d_5/2_ and Pd 3d_3/2_ of the Pd^0^ state in the as‐prepared Co(OH)_2_‐C_3_Pd.^[^ ^41^ ^]^ Two smaller peaks at 336.79 and 342.05 eV correspond to Pd^2+^. The shift to a lower binding energy of Pd may arise from the interaction of relatively larger size Pd with Co and support in the transformed structures of the HzOR‐treated Co(OH)_2_‐C_3_Pd.^[^ ^38^, ^73^ ^]^ ICP‐OES analysis was conducted to evaluate the bulk concentration of Co and Pd in the hybrid catalysts (Table S3, Supporting Information). Due to the extremely low concentration of Pd in the precursor solution, its presence in Co(OH)_2_‐C_1_Pd could not be traced via ICP‐OES. For simplicity, we can assume that the entire Pd is integrated to Co(OH)_2_ and the corresponding mass of Pd in Co(OH)_2_‐C_1_Pd is ≈2.13 µg cm^−2^. However, an increase in the Pd concentration of the precursor has resulted in an almost proportional increase in the Pd presence of the hybrid catalysts (Figure S25, Supporting Information). Surprisingly, a comparison of the atomic % of Pd and Co elements in Co(OH)_2_‐C_3_Pd obtained from the XPS (surface analysis technique) and ICP‐OES (bulk analysis technique) clearly highlights a significantly higher proportion (≈7.45 times) of Pd presence on the surface of the catalyst (Figure 4i). Since electrocatalysis occurs on the surface, a predominant presence of surface‐limited Pd is expected to be beneficial to synergistically enhance the performance of the catalyst hybrids.

### 2.2 Hydrazine Electrooxidation

Electrocatalytic hydrazine oxidation was investigated in both alkaline and alkaline saline electrolytes with hydrazine using a typical three‐electrode cell. Overall, a comparative electrochemical study regarding Co(OH)_2_ and CoFeLDH electrocatalysts was conducted to i) ascertain the role of Fe toward HzOR under the experimental condition and ii) select the hydroxide as a platform suitable for designing a surface‐confined Pd hybrid catalyst aimed to synergistically maximize HzOR under optimum Pd utilization. Afterward, electrocatalytic performances of Pd‐exchanged hydroxide catalysts were optimized and, finally, a comparative analysis was drawn among both hydroxides and their optimum Pd‐exchanged counterparts under the paradigm of the total electrode activity measurements.^[^ ^74^ ^]^

#### 2.2.1 HzOR Performance in Alkaline Electrolyte

**Figure 5:**
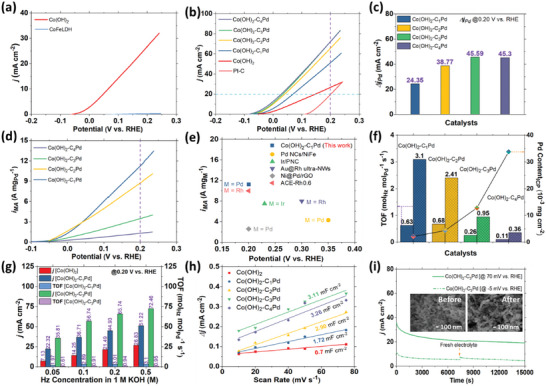
LSV curves of a) Co(OH)_2_ and CoFeLDH, and b) Co(OH)_2_, Co(OH)_2_‐C*_i_* Pd, and Pt—C. LSVs executed at 5 mV s^−1^ in 1.0 m KOH + 0.5 m Hz. c) Contribution of Pd in the HzOR current density at 200 mV. d) Mass activity of Pd hybrids of Co(OH)_2_. e) Mass activity of Pd in Co(OH)_2_‐C_1_Pd measured at 200 mV compared to the reported precious metal catalysts. f) Turnover frequency (TOF) plots generated at 25 mV (with dense line pattern) and 200 mV (with sparse pattern) in conjunction with Pd content obtained from the ICP‐OES analysis for Co(OH)_2_‐C*_i_* Pd. g) Hz concentration dependent current density and TOF values at 0.20 V measured for Co(OH)_2_, Co(OH)_2_‐C_1_Pd, and Co(OH)_2_‐C_3_Pd. h) *C*_dl_ evaluation of Co(OH)_2_ and Co(OH)_2_‐C*_i_* Pd. i) Stability plot of Co(OH)_2_‐C_3_Pd obtained in 1.0 m KOH + 0.5 m Hz with SEM images as inset before and after the test.

Corresponding first LSVs of Co(OH)_2_ and CoFeLDH presented in Figure 1b and Figure S1a, Supporting Information, show higher initial HzOR activity of the former. Subsequent re‐evaluated LSVs in 1 m KOH + 0.5 m Hz are presented in **Figure** **5**a. Co(OH)_2_ performs markedly better toward HzOR as reflected by its low onset potential (defined at 5 mA cm^−2^) of 22 mV and a higher catalytic current density in contrast to CoFeLDH. Co(OH)_2_ demonstrates a current density of 27 mA cm^−2^ at 200 mV; whereas it is 0 mA cm^−2^ at the same potential for CoFeLDH. Therefore, it can be inferred that the presence of Fe inhibits the HzOR activity of Co(OH)_2_. In other words, Fe incorporation to Co(OH)_2_ essentially transforms electrocatalytically active Co(OH)_2_ into a mere support toward HzOR. A relative improvement in current density for Co(OH)_2_ compared to its first LSV could be attributed to the presence of a higher number of surface active Co^2−^ *^*δ*^* sites. Thus, this comparative analysis between Co(OH)_2_ and CoFeLDH reveals that the HzOR activity is element specific although a contrary performance could be expected for a separate anodic process such as OER as opposed to HzOR, where Fe is known for facilitating OER established by numerous research reports.^[^ ^75^, ^76^, ^77^ ^]^ As a result, proper considerations should be taken into account to measure other electrocatalytic parameters of interest apart from LSVs during HzOR. That being the case, Co(OH)_2_ appears not only superior to CoFeLDH toward HzOR but also as the preferred hydroxide to fabricate the optimum Pd catalyst to enhance HzOR to a greater extent.

As‐prepared Pd hybrid of Co(OH)_2_ and CoFeLDH, that is, Co(OH)_2_‐C_1_Pd, Co(OH)_2_‐C_2_Pd, Co(OH)_2_‐C_3_Pd, Co(OH)_2_‐C_4_Pd, and CoFeLDH‐C_3_Pd were also subjected to one linear polarization for the HzOR in 1 m KOH + 0.5 m Hz (Figure 1a and Figure S1a, Supporting Information). An enhanced HzOR activity from all Pd‐exchanged hybrid catalysts is suggestive of the compatibility of Pd with Co in the catalyst design. After this electrochemical pretreatment, the LSVs were re‐assessed in 1 m KOH + 0.5 m Hz (Figure 5b), and HzOR activities of the hybrid catalysts initiate at a further lower potential compared to Co(OH)_2_. This improvement in the onset potential, which is as low as −37 mV, can be credited to the increasing Pd content (Table S3, Supporting Information) in hybrid catalysts, whereas the commercial Pt—C catalyst requires a higher onset potential (145 mV) for HzOR. Similar to the onset potential, Pd exchanged Co(OH)_2_ catalysts also lead to a gradual decrease in the requirement of the working potential to generate a higher HzOR current density, for example, 20 mA cm^−2^. At higher potentials, current densities of Co(OH)_2_‐C_3_Pd and Co(OH)_2_‐C_4_Pd overlap and result in the optimization of the electrocatalytic contribution of Pd. Impressively, Co(OH)_2_‐C_3_Pd only takes a working potential of 200 mV to deliver a HzOR current density of 72.59 mA cm^−2^, the highest among the Co(OH)_2_‐C*_i_* Pd samples. An evaluation of the HzOR current densities at 200 mV for Co(OH)_2_, its Pd hybrids, and commercial Pt—C is also separately presented in Figure S26, Supporting Information, and the realized contribution of Pd in the hybrid catalysts toward HzOR is depicted in Figure 5c. The corresponding current density for commercial Pt—C is significantly lower (19.58 mA cm^−2^) compared to those of Co(OH)_2_‐C*_i_* Pd. In addition, the requirement of the working potentials toward the HzOR reduces to only 33 and 99 mV to deliver 20 and 40 mA cm^−2^, repectively, for Co(OH)_2_‐C_3_Pd compared to corresponding values 1.624 and 1.709 V toward the OER (Figure S27, Supporting Information) presenting the HzOR, in general, as a superior anodic alternative to the high overpotential OER. Another important parameter of interest in examining the electrocatalytic activity is the Tafel slope, a measure of the additional potential required to boost the Faradaic current by an order of magnitude.^[^ ^74^ ^]^ As presented in Figure S28, Supporting Information, the introduction of Pd clearly demonstrates faster HzOR kinetics revealed from the smaller Tafel slopes at ≈*j*_HzOR_ = 10 mA cm^−2^ compared to that of Co(OH)_2_. Co(OH)_2_‐C_3_Pd exhibited the smallest Tafel slope of 103 mV dec^−1^ among Co(OH)_2_ and Pd exchanged hybrid catalysts. HzOR activities of the Pd‐integrated CoFeLDH catalyst, an analogue of Co(OH)_2_‐C_3_Pd are presented in Figure S29, Supporting Information. It highlights that the presence of Fe is not compatible to improve the HzOR activity of Co and Pd resulting in a much lower current density compared to that of Co(OH)_2_ and Co(OH)_2_‐C*_i_* Pd although the atomic percentage of Pd in Co(OH)_2_‐C_3_Pd and CoFeLDH‐C_3_Pd are comparable (Tables S1 and S2, Supporting Information). Overall, the orders of the onset potential and the geometric area normalized current densities at 200 mV for the catalysts can be outlined as follows: *V*_onset_ [Co(OH)_2_‐C*_i_* Pd] < *V*_onset_ [Co(OH)_2_] < *V*_onset_ [CoFeLDH‐C_3_Pd] < *V*_onset_ [CoFeLDH] and *j* [Co(OH)_2_‐C*_i_* Pd] > *j* [Co(OH)_2_] > *j* [CoFeLDH‐C_3_Pd] >> *j* [CoFeLDH].

On a separate note, a closer look at the Pd content in the catalysts (Table S3, Supporting Information) and the HzOR current density highlights that the higher content of Pd has not proportionally contributed to the enhancement of the electrocatalytic activities. It also suggests that an increase in the mass content of Pd essentially contributes to the competitive nature between the number of surface‐active sites and the rise of steric hindrance.^[^ ^38^, ^55^ ^]^ Mass normalized activities of Co(OH)_2_‐C*_i_* Pd are displayed in Figure 5d. Although geometric surface area normalized Co(OH)_2_‐C_3_Pd appears as the optimum under the total electrode measurement, Co(OH)_2_‐C_1_Pd exhibits the highest mass activity of 11.24 A mg_Pd_^−1^ at 200 mV. We would also note that, to date, this is the highest mass activity among the reported values toward HzOR in the alkaline electrolyte as shown in Figure 5e (also detailed in Table S4, Supporting Information, for the comparison). In our system, we observe a decrease in mass activity with higher Pd content suggesting atomic scale Pd with Co(OH)_2_ is critical for obtaining higher mass activity. It is also reflected in the turnover frequency (TOF) values^[^ ^78^, ^79^ ^]^ obtained at 25 and 200 mV for Pd in Co(OH)_2_‐C*_i_* Pd during HzOR in correlation with the Pd content measured from the ICP‐OES analysis as shown in Figure 5f.

We have so far established that the morphological transformation of Co(OH)_2_‐C*_i_* Pd and Co(OH)_2_ is dependent on the concentration of Pd in Co(OH)_2_‐C*_i_* Pd in correlation with the electrocatalytic treatment in 1 m KOH + 0.5 m Hz as shown in Figure 1c, Figure 2a–j, and Figure S3a,b, Supporting Information. Figure 3 and Figures S8–S12, Supporting Information, also illustrate that the structural evolution of Co(OH)_2_‐C_3_Pd as the representative of Co(OH)_2_‐C*_i_* Pd and Co(OH)_2_ is independent of the electrocatalytic HzOR. Apparently, this phenomenon depends on the concentration of hydrazine in addition to Pd and the duration of exposure of the as‐prepared catalysts. Irrespective of such transformation, we notice in Figure 5b,d that the geometric surface area normalized current density of Co(OH)_2_‐C*_i_* Pd and the mass activity of Pd tend to maintain an inverse relation. This correlation further motivated us to explore the structure–activity relationship among Co(OH)_2_, Co(OH)_2_‐C_1_Pd, and Co(OH)_2_‐C_3_Pd dependent on the hydrazine concentration during the HzOR. HzOR activities of Co(OH)_2_‐C_3_Pd, Co(OH)_2_‐C_1_Pd, and Co(OH)_2_ measured at various concentrations of Hz are presented in Figure S30a,b, and S30c, Supporting Information, respectively. No significant anodic current is observed in the absence of hydrazine for geometric surface area optimized Co(OH)_2_‐C_3_Pd. Current densities exhibit a continuous increase with increasing Hz concentration.

Hz concentration dependent TOF plots of Co(OH)_2_‐C_3_Pd and Co(OH)_2_‐C_1_Pd are illustrated in Figure S31a and S31b, Supporting Information, respectively. Surprisingly, we observe that an apparent increase in the current density does not proportionally lead to the enhancement of the TOF values for both Co(OH)_2_‐C_1_Pd and Co(OH)_2_‐C_3_Pd. In order to gain further insight, hydrazine concentration dependent current densities and TOF values obtained at 0.20 V for both Co(OH)_2_‐C_1_Pd and Co(OH)_2_‐C_3_Pd in correlation with corresponding current density of Co(OH)_2_ are presented in Figure 5g. It highlights that although the current density gradually increases with increasing concentration of Hz in the electrolyte, the rate of improvement of the TOF values of Co(OH)_2_‐C_1_Pd and Co(OH)_2_‐C_3_Pd is significantly reduced at Hz concentration >0.1 m Hz in 1 m KOH. Therefore, hydrazine (≤0.1 m) is preferentially electrocatalyzed by metallic Pd, while the contribution of Co(OH)_2_ in the hybrids is significantly improved at higher hydrazine concentration (>0.1 m). In order to correlate this observation, hydrazine concentration dependent electrocatalytically treated Co(OH)_2_‐C_1_Pd and Co(OH)_2_‐C_3_Pd samples were also characterized by SEM. Figure S32, Supporting Information, shows that structurally morphed Co(OH)_2_‐C_3_Pd appears to remain broadly unchanged in correlation to hydrazine concentration and the corresponding image at Figure 2j. However, a gradual morphological change along with the coexistence of the layered structure of Co(OH)_2_‐C_1_Pd compared to that of as presented in Figure 2h is featured with increasing concentration of hydrazine (Figure S33, Supporting Information). It is also in line with the SEM observation as presented in Figure S13, Supporting Information. Although hydrazine induced transformed Co(OH)_2_ is structurally different in absence of Pd (Figure S12, Supporting Information), surface confined metallic Pd is the dominant catalyst irrespective of the morphed structure of Co(OH)_2_‐C*_i_* Pd.

Electrocatalytic activities of different catalysts are normally correlated with the corresponding electrochemical surface area in relation to the double‐layer capacitance (*C*_dl_) shown in Figure 5h. *C*_dl_ values are obtained from the cyclic voltammograms generated at different scan rates (Figure S34a–e, Supporting Information). Co(OH)_2_‐C_3_Pd and Co(OH)_2_‐C_4_Pd show comparably higher *C*_dl_ values of 3.11 and 3.26 mF cm^−2^, respectively, reflecting their HzOR activities arising from the higher number of active sites. On the other hand, the Co(OH)_2_ demonstrates a significantly smaller *C*_dl_ value of 0.7 mF cm^−2^. Therefore, the Pd hybrid catalysts of Co(OH)_2_ offer a higher electrochemical surface area that leads to synergistically improved HzOR activities. The electrochemical impedance spectroscopy (EIS) tests were also performed (Figure S35a, Supporting Information) and show Pd induced smaller charge transfer resistance for Co(OH)_2_‐C_3_Pd compared to that of Co(OH)_2_. A decrease in the phase angle for Co(OH)_2_‐C_3_Pd in the lower frequency region of the Bode phase plots (Figure S35b, Supporting Information) is related to HzOR.^[^ ^38^ ^]^ As shown in the stability plot of Co(OH)_2_‐C_3_Pd in Figure 5i, a gradual decline of *j* at −5 and 70 mV in 1 m KOH + 0.5 m Hz could be due to i) the relatively reduced diffusion of Hz from the bulk to the electrode surface, ii) the continuous decrease in the concentration of Hz over time, iii) the detachment of some active catalysts from the electrode, and iv) the blockage of active sites due to the generation of gas bubbles. Meanwhile, exposure to the fresh electrolyte again shows similar electrocatalytic performance. Post‐stability SEM images (inset) reveal no noticeable change in the morphology. XPS analysis was also conducted to detect the probable changes of the surface properties of Co(OH)_2_‐C_3_Pd after the stability test. Survey spectrum (Figure S36a, Supporting Information) shows the presence of Co, Pd, and O. Two peaks at 780.81 and 796.83 eV in the Co 2p (Figure S36b, Supporting Information) can be assigned to Co 2p_3/2_ and Co 2p_1/2_ of Co(OH)_2_, respectively;^[^ ^80^ ^]^ whereas, corresponding peak binding energies of Pd 3d_5/2_ and Pd 3d_3/2_ as presented in Figure S36c, Supporting Information, indicate the existence of Pd^0^.^[^ ^41^ ^]^ However, an apparent shift of Pd 3d and Co 2p peaks to the higher binding energy suggests partial oxidation; it could be due to KOH in the electrolyte and/or associated with XPS operation. Continuous gas evolution on the electrode surface may lead to the detachment of a portion of structurally transformed Co(OH)_2_‐C_3_Pd and, thereby, reduced peak intensities of Co 2p and Pd 3d.

#### 2.2.2 Hydrazine Electrooxidation in Alkaline Saline Electrolyte

The importance of examining HzOR in the alkaline saline electrolyte (1 m KOH + 0.5 m NaCl) is associated with the vastness of ocean water as an alternative energy source. Building an electrochemical device, for example, water splitting in alkaline seawater at high current densities is extremely challenging due to the competitive nature of the anodic OER and ClOR (depicted in Figure 1a). HzOR as an anodic reaction alternative could be beneficial in realizing counter‐cathodic reactions at low electrochemical cell potentials while corrosive reactions to the electrodes initiated by chlorine are particularly avoided. Therefore, we also systematically examined the HzOR performances of the aforementioned catalysts in 1 m KOH + 0.5 m NaCl with a certain amount of Hz.

**Figure 6:**
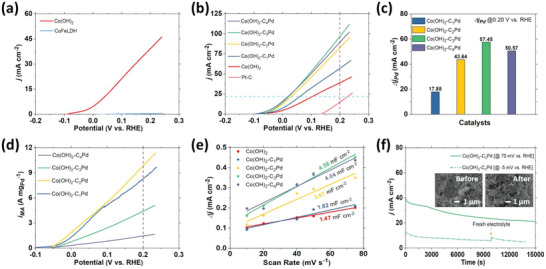
LSV curves of a) Co(OH)_2_ and CoFeLDH, and b) Co(OH)_2_, Co(OH)_2_‐C*_i_* Pd, and Pt—C. LSVs obtained at 5 mV s^−1^ in 1.0 m KOH + 0.5 NaCl + 0.5 m Hz. Contribution of Pd in HzOR c) current density at 200 mV and d) mass activity. e) *C*_dl_ evaluation of Co(OH)_2_ and Co(OH)_2_‐C*_i_* Pd. f) Stability plot of Co(OH)_2_‐C_3_Pd in 1.0 m KOH + 0.5 NaCl + 0.5 m Hz with SEM images as insets before and after the test.

All as‐prepared catalysts were treated with one linear polarization in 1 m KOH + 0.5 m NaCl + 0.5 m Hz (Figure 1d and Figure S1b, Supporting Information) and gently washed with ethanol followed by air drying before the determination of the relevant electrocatalytic properties of interest. The reassessed polarization curves of Co(OH)_2_ and CoFeLDH (**Figure** **6**a) confirm the prevailing outperformance of Co(OH)_2_ as an individual catalyst, while its improved current density compared to the first polarization also aligns with the earlier observation in alkaline HzOR. Co(OH)_2_ exhibits not only a requirement of smaller onset potential but also an overall high current density surpassing its corresponding values observed in 1 m KOH + 0.5 m Hz.

Linear polarization curves and corresponding Tafel plots toward HzOR of Co(OH)_2_, Co(OH)_2_‐C*_i_* Pd, and Pt—C catalysts are presented in Figure 6b and Figure S37, Supporting Information, respectively. The parameters of interest such as onset potential, overpotential requirement, and Tafel slope show a gradual improvement in the electrocatalytic properties resulting from the integration of Pd with Co(OH)_2_. The trend of the HzOR onset potential is as follows: *V*_onset_ [Co(OH)_2_‐C*_i_* Pd] < *V*_onset_ [Co(OH)_2_] < *V*_onset_ [Pt—C]. The Pd‐integrated Co(OH)_2_ exhibits an onset potential as low as −47 mV compared to that of Co(OH)_2_ at −3 mV and Pt—C at 159 mV. Excellent HzOR activities at higher potentials amplify the synergistic contribution between Co and Pd. Co(OH)_2_‐C_3_Pd demonstrates the smallest Tafel slope of 81 mV dec^−1^ indicating faster HzOR kinetics.^[^ ^15^ ^]^ Therefore, it is clear that the addition of NaCl in 1 m KOH + 0.5 m Hz has been beneficial in enhancing the HzOR activities of the catalysts. Song et al.^[^ ^63^ ^]^ also observed the outperformance of OER in alkaline saline water compared to that of pure alkaline electrolytes. While there are visible improvements in the HzOR activities of the CoFeLDH‐C_3_Pd compared to the CoFeLDH (Figure S38, Supporting Information), electrocatalytic performance remains unsatisfactory relative to both Co(OH)_2_ and Co(OH)_3_‐C*_i_* Pd. Overall, geometric area normalized current densities at 200 mV also follow the earlier mentioned trend: *j* [Co(OH)_2_‐C*_i_* Pd] > *j* [Co(OH)_2_] > *j* [CoFeLDH‐C_3_Pd] >> *j* [CoFeLDH]. Evidently, Co(OH)_2_‐C_3_Pd exhibits an outstanding current density of 96.67 mA cm^−2^ at 200 mV, which is not only the best among the hybrids but also 2.46 and 6.48 times that of Co(OH)_2_ and Pt—C, respectively (Figure S39, Supporting Information). The realized electrocatalytic contribution of Pd for its hybrid catalysts with Co(OH)_2_ toward HzOR is presented in Figure 6c. Potential dependent mass normalized activities of Co(OH)_2_‐C*_i_* Pd are displayed in Figure 6d. In the alkaline saline electrolyte with 0.5 m Hz, Co(OH)_2_‐C_2_Pd exhibits the highest mass activity among the catalysts and the corresponding value is 9.83 A mg_Pd_^−1^ at 200 mV. To the best of our knowledge, it is the highest mass activity of any precious metal catalysts obtained at such a low working potential in alkaline saline electrolyte with hydrazine (See Table S4, Supporting Information, for the detailed comparison). Co(OH)_2_‐C_1_Pd also demonstrates a high mass activity of 8.39 A mg_Pd_^−1^. In alignment with earlier observation (Figure 5f), a significant decrease in TOF values at 25 and 200 mV (Figure S40, Supporting Information) in contrast to the geometric surface area normalized high current densities of Co(OH)_3_‐C_3_Pd and Co(OH)_3_‐C_4_Pd also highlights that there may exist a larger scope in realizing atomic scale Pd catalyst for enhanced HzOR activities in simulated alkaline seawater with hydrazine.

As shown in hydrazine concentration‐dependent linear polarizations of Co(OH)_2_‐C_3_Pd (Figure S41, Supporting Information), with incremental hydrazine concentration, HzOR current densities continually increase in successive polarization. Electrocatalytic activities of the catalysts are also correlated with the double‐layer capacitance obtained in saline electrolytes with 0.5 m Hz (Figure 6e). Scan rate dependent cyclic voltammograms of Co(OH)_2_ and Co(OH)_2_‐C*_i_* Pd are presented in Figure S42a–e, Supporting Information. Co(OH)_2_‐C_3_Pd exhibits the highest *C*_dl_ values of 4.58 mF cm^−2^ and corresponds well with its HzOR activities. Interestingly, double‐layer capacitance is found to increase for other catalysts as well in comparison to their analogous values obtained in the absence of 0.5 m NaCl. A relatively higher *C*_dl_ suggests better adsorption and desorption of the charged species on the surface sites facilitating high HzOR in the saline electrolyte. In addition, the EIS spectra and the Bode phase plots of Co(OH)_2_‐C_3_Pd and Co(OH)_2_ as shown in Figure S43, Supporting Information, reflect the observations as shown in Figure S35, Supporting Information. The *j–t* plot of Co(OH)_2_‐C_3_Pd is presented in Figure 6f showing a profile similar to our earlier observation and suggests the operability of the electrode at high or low current densities. SEM images (insets) feature no detectable morphological transformation as well.

#### 2.2.3 Energy Saving Hydrogen Generation in Two‐Electrode Configuration

Superior HzOR performance of Co(OH)_2_‐C*_i_* Pd also led us to explore the HzOR coupled HER process under the two‐electrode configuration with an aim to replace the OER and avoid the ClOR. Prior to this electrochemical characterization, HER activities of Co(OH)_2_ and Co(OH)_2_‐C*_i_* Pd were tested under a three‐electrode configuration in 1 m KOH and 1 m KOH + 0.5 m NaCl. Although Co(OH)_2_‐C*_i_* Pd demonstrates apparently improved HER current densities compared to that of Co(OH)_2_ at high overpotential (Figure S44a,b, Supporting Information), overall performances are significantly lower than that of standard Pt—C electrocatalyst. Therefore, two‐electrode configuration was assembled with Co(OH)_2_‐C_3_Pd (+)||Pt—C (−) as a case study for both overall hydrazine splitting (OHzS) and overall water splitting (OWS). As shown in Figure S44c, Supporting Information, Co(OH)_2_‐C_3_Pd driven OHzS electrolyzer requires only 0.095 and 0.221 V to generate a current density of 10 and 20 mA cm^−2^, respectively, in 1 m KOH + 0.5 m Hz while generating hydrogen at Pt—C electrode. The corresponding values for the OWS are 1.692 and 1.842 V. Similarly, the requirement of the electrochemical cell potential for the HzOR enabled HER in 1 m KOH + 0.5 m NaCl + 0.5 m Hz (Figure S44d, Supporting Information) is lowered by 1.571 and 1.558 V in order to generate an anodic current density of 10 and 20 mA cm^−2^, respectively. As a result, the reduced operating cell potential for hydrogen generation reaffirms the thermodynamic advantages of the hydrazine assisted hydrogen generation.

## 3 Conclusion

In summary, we have successfully demonstrated that the ultra‐low scale Pd confined on the Co(OH)_2_ surface is a competent catalyst to synergistically enhance HzOR in both alkaline and alkaline saline electrolytes as an alternative to the conventional OER and the corrosive ClOR. To select Co(OH)_2_ as a desired platform for HzOR, a comparative analysis between Co(OH)_2_ and CoFeLDH is presented. The Pd hybrids of Co(OH)_2_ were synthesized by integrating Pd into electrodeposited layered Co(OH)_2_ via an in situ redox process at room temperature. SEM characterization confirms that Co(OH)_2_ and its Pd hybrids were transformed with the shape of nanoparticles of irregular geometry from their corresponding layered structures during HzOR. However, it has been systematically established that this feature of morphological transformation is independent of electrocatalytic HzOR; rather, it depends on the Pd content in Co(OH)_2_, the concentration of hydrazine in the solution, and the duration of exposure. The presence of Fe in the catalysts suppresses the inherent HzOR activity of both Co in Co(OH)_2_ and Pd. On the other hand, surface confinement of Pd with Co(OH)_2_ results in the synergistic improvement in HzOR with lower onset potentials, significantly higher current densities, smaller Tafel slopes, and immense mass activities. The mass activities of Pd are 11.24 and 9.83 A mg_Pd_^−1^ at 200 mV in alkaline and alkaline saline electrolytes with 0.5 m Hz, respectively. To date, these are the highest HzOR mass activities among all precious metal‐based catalysts in the respective electrolyte when compared to the reported values in the literature (Table S4, Supporting Information). Geometric surface area normalized Co(OH)_2_‐C_3_Pd also shows a high current density relative to commercial Pt—C and a fairly stable HzOR performance. In addition, structure‐activity relationship reveals preferential electrocatalytic HzOR on metallic Pd in Co(OH)_2_‐C*_i_* Pd. Finally, this study guides the proper selection of the platform as well as the design of surface‐limited precious metal catalysts and suggests their broader applications in electrocatalysis and beyond.

## 4 Experimental Section

### Chemicals

Carbon cloth (CC, HCP331; 0.35 +/−0.02 mm; WizMAC Co. Ltd.), Co(NO_3_)_2_·6H_2_O (≥98%, ACS reagent; Sigma‐Aldrich), FeSO_4_·7H_2_O (≥99%; Sigma‐Aldrich), hydrazine monohydrate (80% Assay; Daejung Chemicals), KOH (85% Assay, Extreme pure; Reagents Duksan), anhydrous ethanol (99.5%; Daejung Chemicals), Na_2_PdCl_4_ (98%; Sigma‐Aldrich), NaCl (≥99% ACS reagent; Sigma‐Aldrich), Pt—C (20 wt% on carbon black; Alfa Aesar). All chemicals were used as received unless a specific process or purification was mentioned separately.

### Preparation of the Electrodes on Carbon Cloth

Layered Co(OH)_2_ and CoFeLDH were prepared by a single‐step electrochemical deposition.^[^ ^81^, ^82^ ^]^ Before electrodeposition, pieces of carbon cloth were washed with ethanol under ultrasonication for 10 min and later, dried in an electric oven at 80°C for 5 min. Electrodeposition was carried out at an electrochemical workstation (VersaSTAT4 Potentiostat, AMETEK Princeton Applied Research). For a typical synthesis of layered Co(OH)_2_, a piece of CC (≈2 × 0.8 × 0.8 cm^2^) was exposed in an aqueous electrolyte made of 0.04 m 40 mL Co(NO_3_)_2_·3H_2_O.^[^ ^83^ ^]^ A constant charge of −1.4C was applied under a three‐electrode system, where Pt mesh, Ag/AgCl (3.4 m KCl), and CC were used as the counter, the reference, and the working electrode, respectively. The electrodeposited samples were gently washed in ethanol and air‐dried. CoFeLDH was similarly electrodeposited on CC for a constant charge of −1.4C utilizing 0.04 m 40 mL electrolyte made of an equimolar composition of Co(NO_3_)_2_·H_2_O and FeSO_4_·7H_2_O. CoFe LDH samples were stored in and washed with ethanol before drying in the electric oven for 3 min at 80 °C.

### Pd Incorporation to Co(OH)_2_ and CoFeLDH via In Situ Redox Process

A 2.5 mm stock solution of Pd was prepared by dissolving Na_2_PdCl_4_ in anhydrous ethanol. The redox process was conducted for different concentrations (0.01, 0.05, 0.10, and 0.20 mm 4 mL) of Pd precursors in a glass vial for 24 h under the dark condition at room temperature. Later, samples were collected and washed in ethanol, and dried in air. Pd integrated samples of Co(OH)_2_ were designated by Co(OH)_2_‐C*_i_* Pd. Here, *i* refers to various Pd exchanged samples: Co(OH)_2_‐C_1_Pd, Co(OH)_2_‐C_2_Pd, Co(OH)_2_‐C_3_Pd, and Co(OH)_2_‐C_4_Pd, where C_1_, C_2_, C_3_, and C_4_ stand for 0.01, 0.05, 0.10, and 0.20 mm 4 mL Pd solution, respectively. CoFeLDH‐C_3_Pd was also obtained similarly.

### Coating of Pt—C on CC

10 mg of Pt—C was added to a 2 mL solution of DI water, absolute ethanol, and Nafion, where the respective volumetric ratio was 15:4:1. A homogeneous ink was prepared via ultrasonication for 20 min. Pt—C catalyst was coated on a piece of CC by dipping it in the ink. Afterward, the as‐coated sample was air‐dried before electrochemical characterization.

### Physical Characterization

Morphological characterization was performed with SEM (JSM‐7600F, JEOL) at an accelerating voltage and the EDX analysis was carried out at 15 kV. TEM measurements and corresponding elemental mapping were conducted with the Titan G2 ChemiSTEM Cs Probe (ThermoFisher Scientific, USA) at 200 kV. The crystalline structure of the samples was evaluated with an automated multipurpose XRD (Rigaku SmartLab) operated at 45 kV and 200 mA. Pd and Co content in the catalysts were probed by an inductively coupled plasma‐optical emission spectroscopy (OPTIMA 8300, Perkin‐Elmer, USA). The chemical composition and the valence states were evaluated using XPS (NEXSA, ThermoFisher Scientific, USA).

### Electrochemical Measurements

Electrochemical measurements were performed with a standard three‐electrode system at an electrochemical workstation (VersaSTAT4 Potentiostat, AMETEK Princeton Applied Research) at room temperature. CC‐supported catalysts (≈2 × 0.8 × 0.4 cm^2^) were used as the working electrode, whereas Hg/HgO (1.0 m NaOH) and Pt mesh were used as the reference and the auxiliary electrode, respectively. All measured potentials were converted to the RHE scale

(4) $$E\;(\mathrm{vs}\;\mathrm{RHE})=E_{\frac{\mathrm{Hg}}{\mathrm{HgO}}}+0.0591\times \mathrm{pH}+E_{\frac{\mathrm{Hg}}{\mathrm{HgO}}}^{o}$$

All potential values referred to here were measured against the RHE unless specified. Hydrazine electrooxidation was carried out in the alkaline (saline) electrolytes: aqueous 1 m KOH (pH ≈ 14) and 1 m KOH + 0.5 m NaCl (pH ≈ 14) with a certain concentration of hydrazine (Hz). This electrocatalytic study was subject to 0.5 m as the maximum concentration of N_2_H_4_. LSV curves were acquired at a scan rate of 5 mV s^−1^ to reduce background capacitive current and recorded without *iR* compensation. Tafel slopes were obtained from the LSV curves according to the Tafel equation, *η* = *b* log*|j| + a*, where *η*, *b*, *j*, and *a* represent overpotential, Tafel slope, current density, and Tafel constant, respectively. First, as part of broader measurements of the electrocatalysis, all as‐prepared catalysts were initiated with an electrochemical treatment toward HzOR with one LSV from −1.1 to −0.70 V versus Hg/HgO at 5 mV s^−1^ in both alkaline and alkaline saline electrolyte mixed with 0.5 m Hz to ascertain the structural integrity of the catalysts in a reductive environment and obtain stabilized electrocatalytic performances. TOF for Pd catalyzed electrochemical oxidation of hydrazine was calculated based on the equation: $\mathrm{TOF}\;=\frac{\Delta j}{4Fn_{\mathrm{Pd}}}$.^[^ ^79^ ^]^ Here, *∆j*, *F*, and *n*_Pd_ are Pd catalyzed current density, Faraday constant (96 485 C mol^−1^), and moles of Pd per unit area. In order to realize hydrazine concentration‐dependent electrocatalytic behavior, LSVs were evaluated for various concentrations of hydrazine (0, 0.05, 0.1, 0.2, and 0.5 m) maintaining 90 s delay between successive polarization for addition and uniform distribution of N_2_H_4_ in the alkaline (saline) electrolyte. Electrocatalytic performances were correlated to the electrochemical double‐layer capacitance (*C*_dl_). Cyclic voltammograms were generated at various scan rates in the potential window of −0.975 to −1.025 V (vs Hg/HgO). The current density differences [*Δj = |j*_a_ *− j*_c_ *|/2*] were plotted against the scan rates and the obtained value of the linear slope was *C*_dl_.^[^ ^84^ ^]^ Impedance spectroscopic measurements were carried out at −0.975 V versus Hg/HgO. A chronoamperometric stability test was performed at −0.95 and −0.875 V (vs Hg/HgO). HER performance was evaluated under the three‐electrode configuration with Pt foil and graphite as the counter electrode in 1 m KOH and 1 m KOH + 0.5 m NaCl, respectively.

## Conflict of Interest

The authors declare no conflict of interest.

## Supporting information

Supporting Information

## Data Availability

Research data are not shared.
