# Supplementary material for: Surface‐Confined Ultra‐Low Scale Pd Engineered Layered Co(OH)2 toward High‐Performance Hydrazine Electrooxidation in Alkaline Saline Water
Source: Adv Sci (Weinh). 2023 Apr 29;10(21):2300639. doi: 10.1002/advs.202300639 (PMC10375158; doi:10.1002/advs.202300639)
Supplement: Supplementary file 1 — Supporting Information [file ADVS-10-2300639-s001.pdf]

ADVANCED SCIENCE
Open Access

Supporting Information

for *Adv. Sci.*, DOI 10.1002/advs.202300639

Surface-Confined Ultra-Low Scale Pd Engineered Layered $Co(OH)_2$ toward High-Performance Hydrazine Electrooxidation in Alkaline Saline Water

*Swagotom Sarker, Ji Hoon Choi, Hak Hyeon Lee, Dong Su Kim and Hyung Koun Cho**

Supporting Information

Surface-Confined Ultra-low Scale Pd Engineered Layered $Co(OH)_2$ towards High-Performance Hydrazine Electrooxidation in Alkaline Saline Water

Swagotom Sarker,[a,1] *Ji Hoon Choi,*[a,1] *Hak Hyeon Lee,*[a] *Dong Su Kim,*[a] *and Hyung Koun Cho*[a,]*

[a] School of Advanced Materials Science and Engineering, Sungkyunkwan University (SKKU), 2066, Seobu-ro, Jangan-gu, Suwon-si, Gyeonggi-do 16419, Republic of Korea
E-mail: chohk@skku.edu

[1] Swagotom Sarker and Ji Hoon Choi contributed equally to this work.

Number of pages: 27

Number of figures: 44

Number of tables: 4

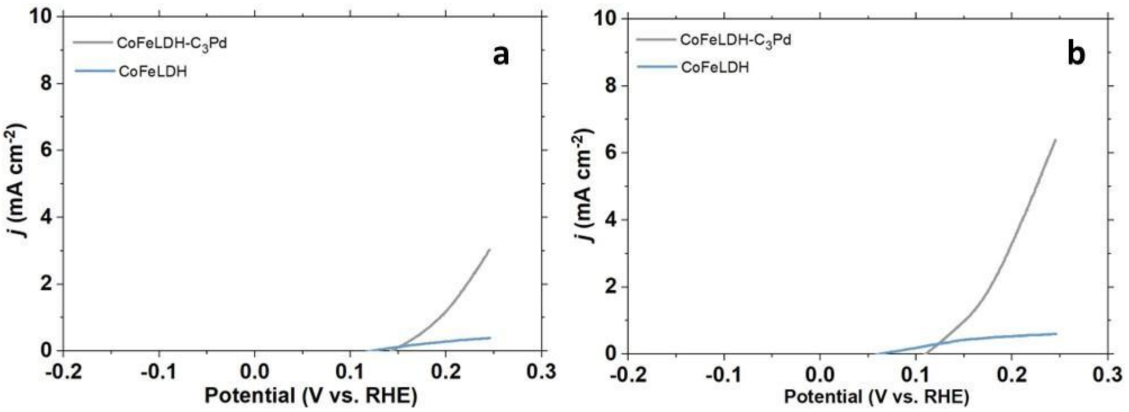

Figure S1. 1st LSV curves of CoFeLDH and its Pd hybrid generated at 5 mV s^{-1} in 1 M KOH + 0.5 M Hz (a) and 1 M KOH + 0.5 M NaCl + 0.5 M Hz (b).

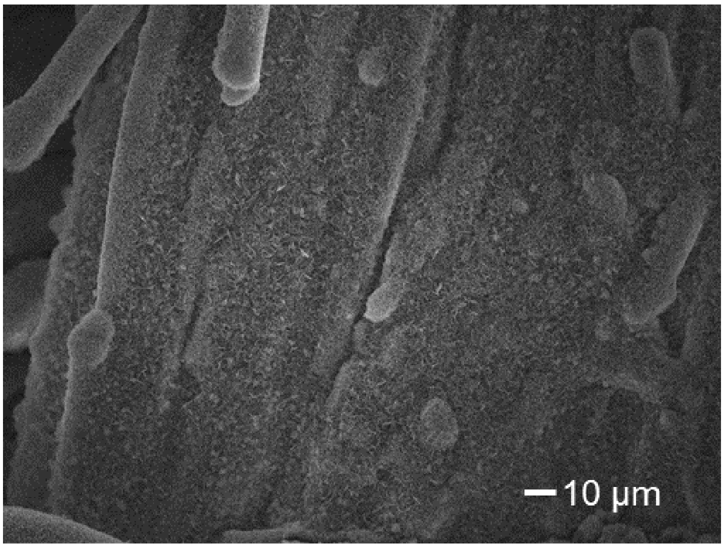

Figure S2. SEM image of as-prepared $Co(OH)_2$ grown on carbon cloth.

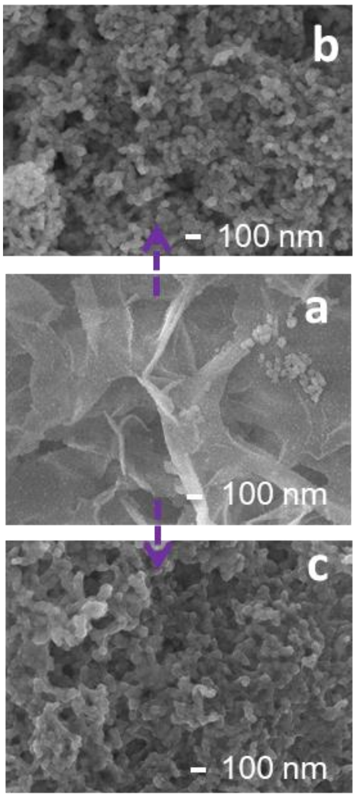

Figure S3. SEM images of as-prepared $Co(OH)_2$-C_4Pd (a) with corresponding HzOR treated sample images after 1st LSV at 5 mV s^{-1} in 1 M KOH + 0.5 M Hz (b) and 1 M KOH + 0.5 M NaCl + 0.5 M Hz (c). Arrow (→) is used to correlate the image of the as-prepared sample to HzOR-treated samples.

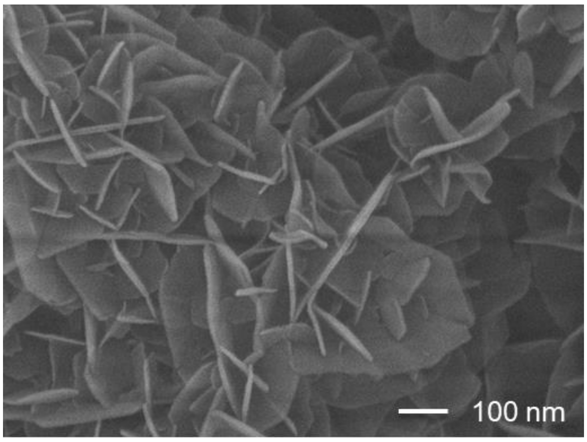

Figure S4. SEM image of as-prepared CoFeLDH-C_3Pd.

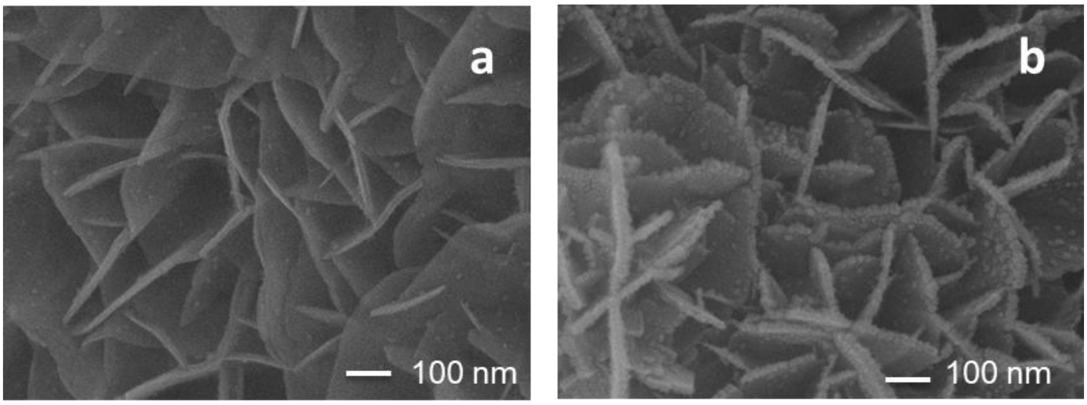

Figure S5. SEM images of CoFeLDH-C_3Pd after 1st LSV at 5 mV s^{-1} in 1 M KOH + 0.5 M Hz (a) and 1 M KOH + 0.5 M NaCl + 0.5 M Hz (b).

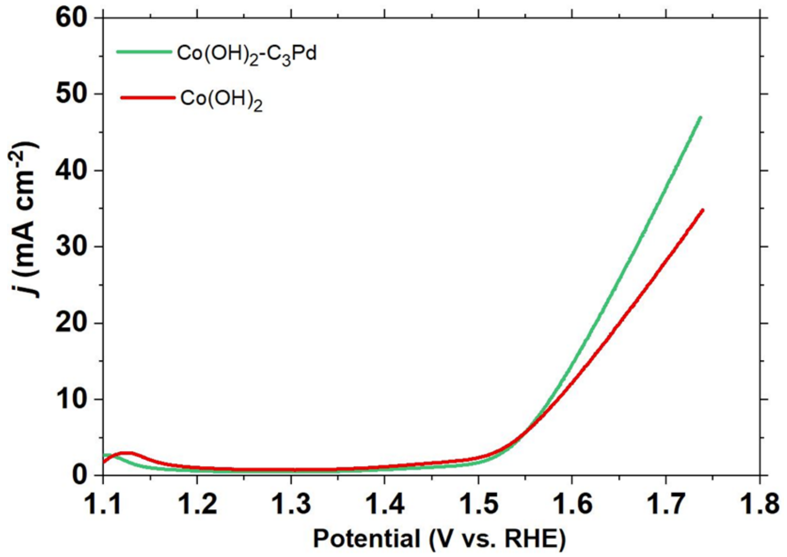

Figure S6. LSV generated at 5 mV s^{-1} towards OER in 1 M KOH for $Co(OH)_2$ and $Co(OH)_2$-C_3Pd.

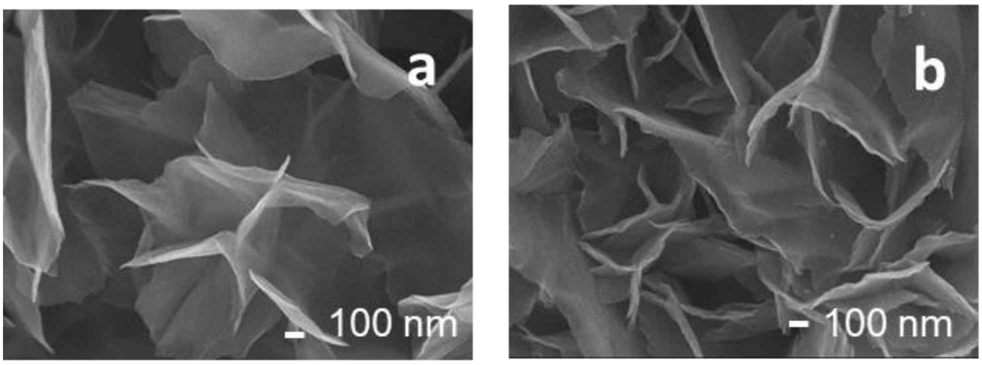

Figure S7. SEM images of $Co(OH)_2$ (a) and $Co(OH)_2$-C_3Pd (b) after OER treatment in 1 M KOH.

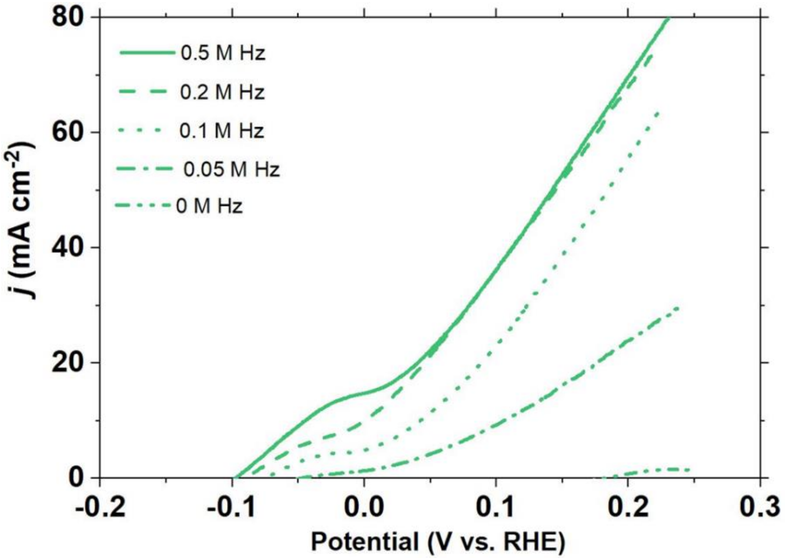

Figure S8. 1st LSV generated at 5 mV s^{-1} towards HzOR in 1 M KOH with 0 M, 0.05 M, 0.1 M, 0.2 M, and 0.5 M Hz for as-prepared $Co(OH)_2$-C_3Pd.

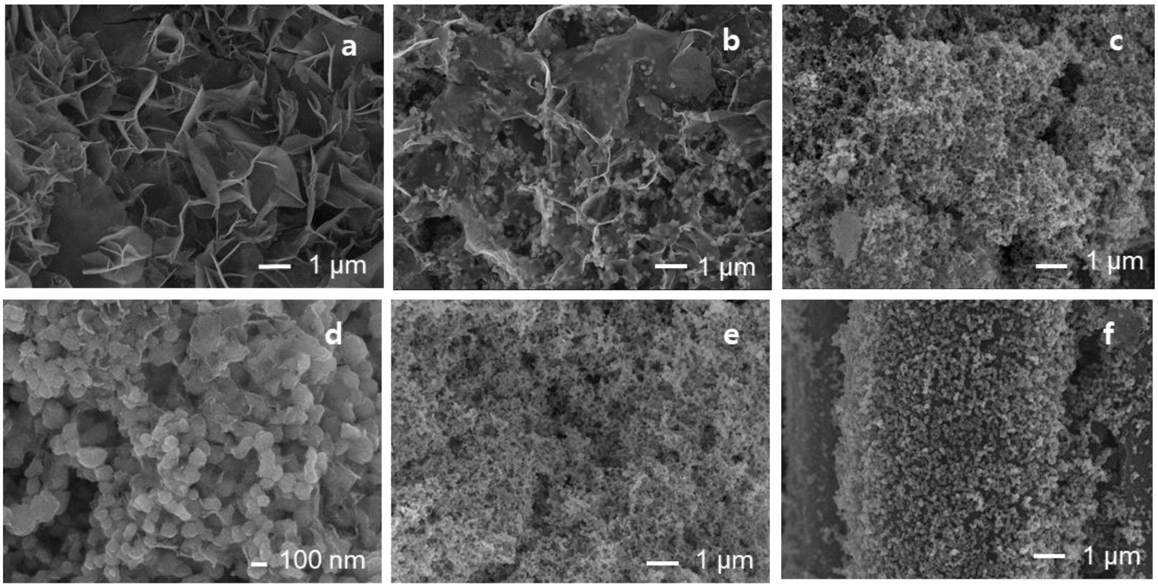

Figure S9. Low-resolution SEM images of $Co(OH)_2$-C_3Pd after 1st LSV at 5 mV s^{-1} towards HzOR in 1 M KOH with 0 M (a), 0.05 M (b), 0.1 M (c), 0.2 M (d), and 0.5 M Hz (e). SEM image of $Co(OH)_2$-C_3Pd after exposure for 80 s in 1 M KOH + 0.5 M Hz (f).

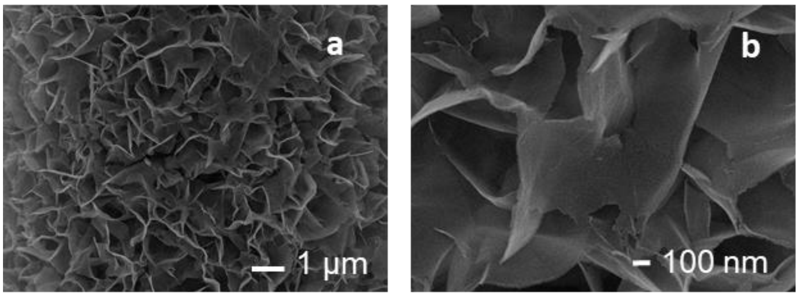

Figure S10. SEM images of $Co(OH)_2$ after exposure for 80 s in 1 M KOH + 0.5 M Hz.

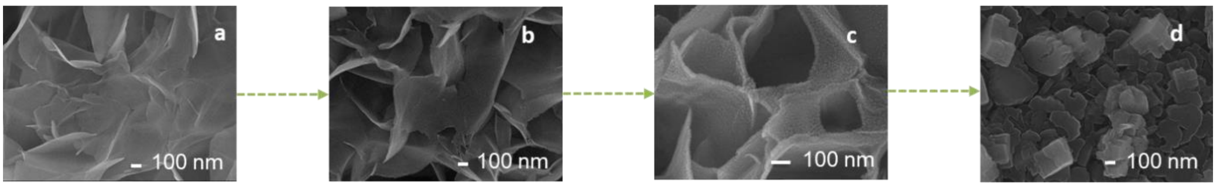

Figure S11. SEM images of $Co(OH)_2$ after exposure for 0 s i.e. as-prepared sample (also shown in **Figure 2a**) (a), 80 s (b), 480 s (c), and 1600 s (d) in 1 M KOH + 0.5 M Hz.

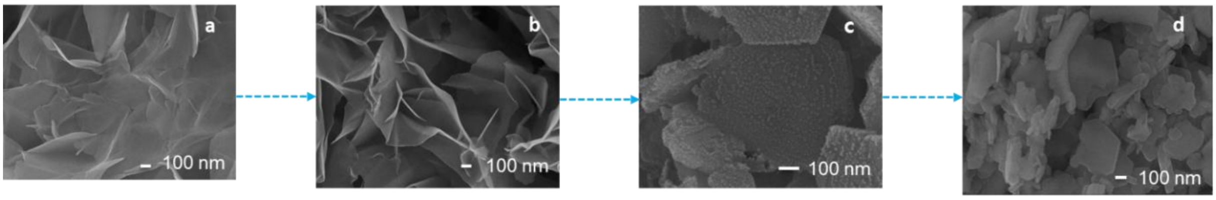

Figure S12. SEM images of $Co(OH)_2$ after different numbers of LSV executed from -1.1 to -0.70 V vs. Hg/HgO at 5 mV s^{-1} towards HzOR in 1 M KOH + 0.5 M Hz. Number of LSVs: 0 i.e. as-prepared (also shown in **Figure 2a**) (a), 1 (also shown in **Figure 2f**) (b), 6 (c), and 20 (d).

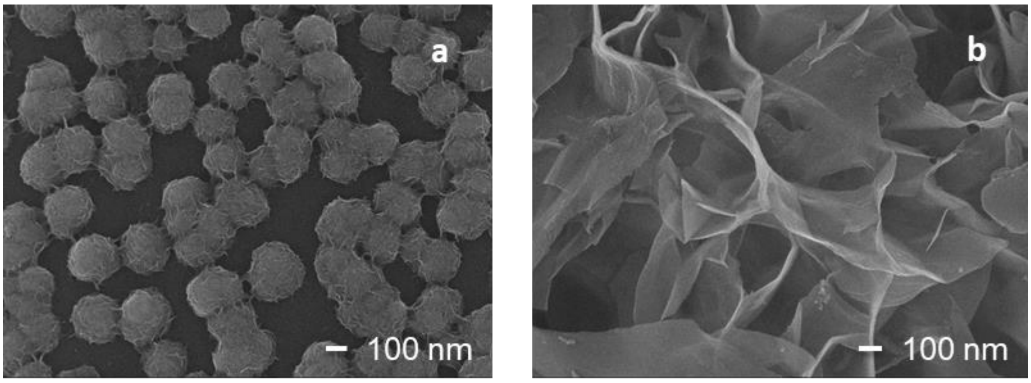

Figure S13. SEM images of $Co(OH)_2$-C_1Pd after HzOR treatment in 1 M KOH + 0.5 M Hz (a, b). Number of LSVs executed at 5 mV s^{-1} from -1.1 to -0.70 V vs. Hg/HgO: 3.

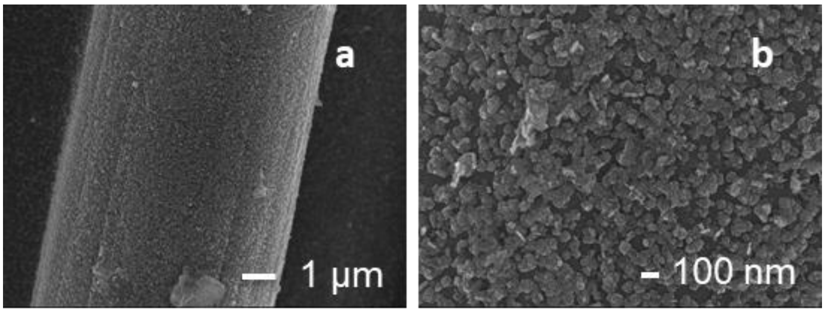

Figure S14. SEM images of CoFeLDH-C_3Pd after HzOR treatment in 1 M KOH + 0.5 M Hz (a, b). Number of LSVs executed at 5 mV s^{-1} from -1.1 to -0.70 V vs Hg/HgO: 20.

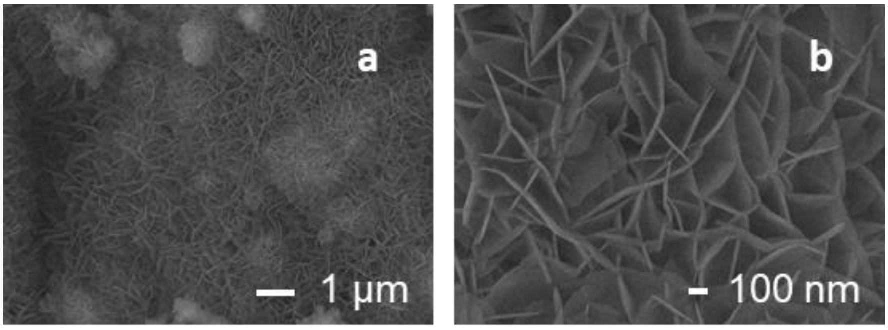

Figure S15. SEM images of CoFeLDH after HzOR treatment in 1 M KOH + 0.5 M Hz (a, b). Number of LSVs executed from -1.1 to -0.70 V vs. Hg/HgO at 5 mV s^{-1}: 20.

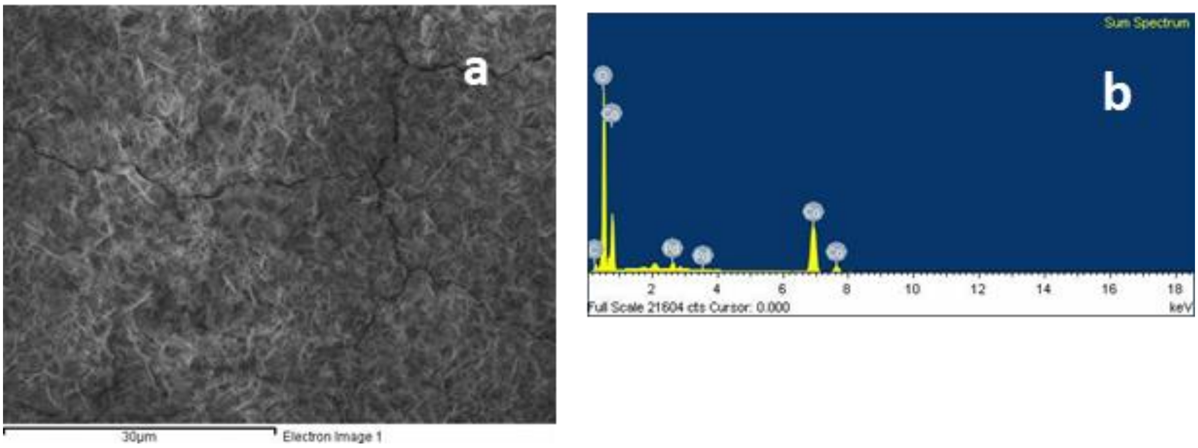

Figure S16. EDX pattern of as-prepared $Co(OH)_2$-C_3Pd (a, b).

Table S1. Elemental composition of as-prepared $Co(OH)_2$-C_3Pd obtained from EDX analysis.

Element	Wt%	Atomic%
C K	4.54	10.15
O K	38.21	64.10
Co K	55.65	25.34
Pd L	1.60	0.40

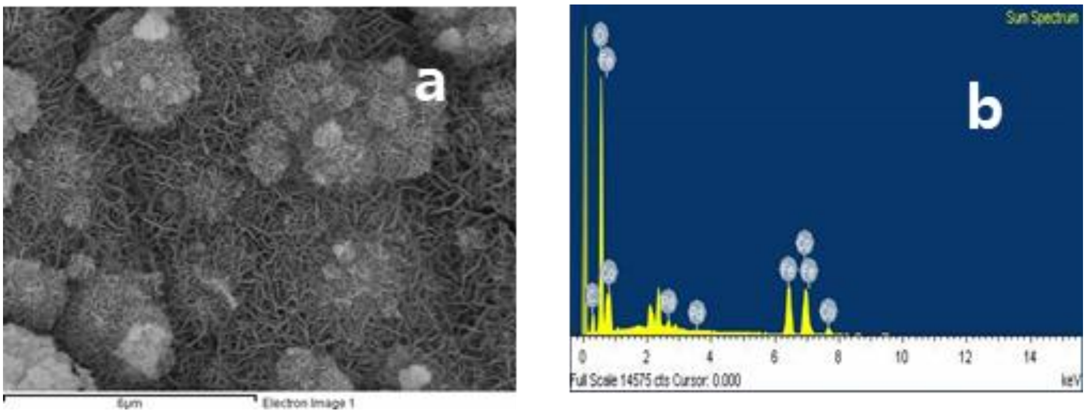

Figure S17. EDX pattern of as-prepared CoFeLDH-C_3Pd (a, b).

Table S2. Elemental composition of as-prepared CoFeLDH-C_3Pd obtained from EDX analysis.

Element	Wt%	Atomic%
C K	5.75	13.24
O K	33.57	57.98
Co K	32.57	15.27
Fe	26.40	13.06
Pd L	1.71	0.44

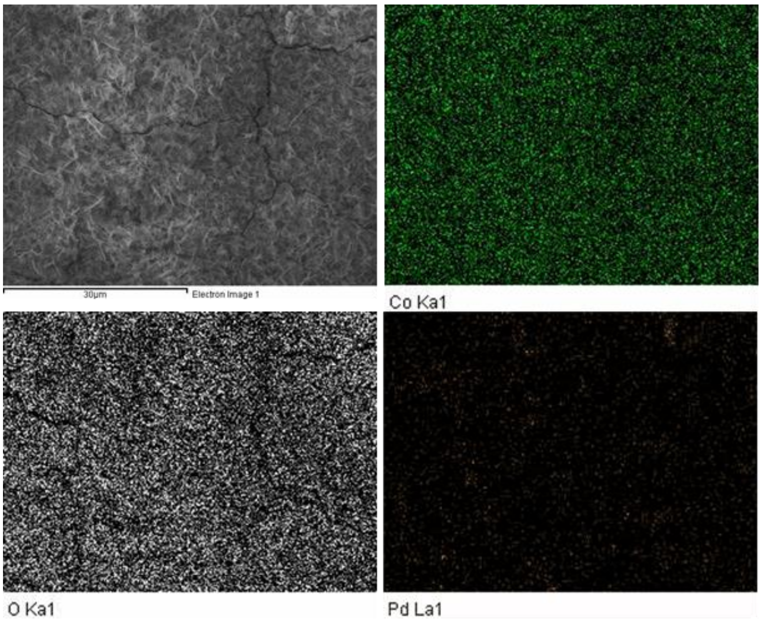

Figure S18. EDX-elemental mapping images of as-prepared $Co(OH)_2$-C_3Pd.

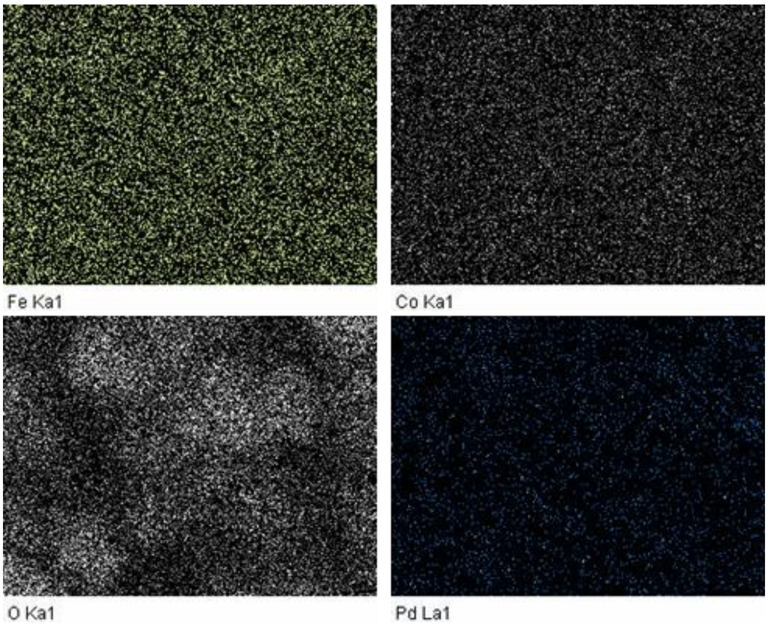

Figure S19. EDX-Elemental mapping images of as-prepared CoFeLDH-C_3Pd.

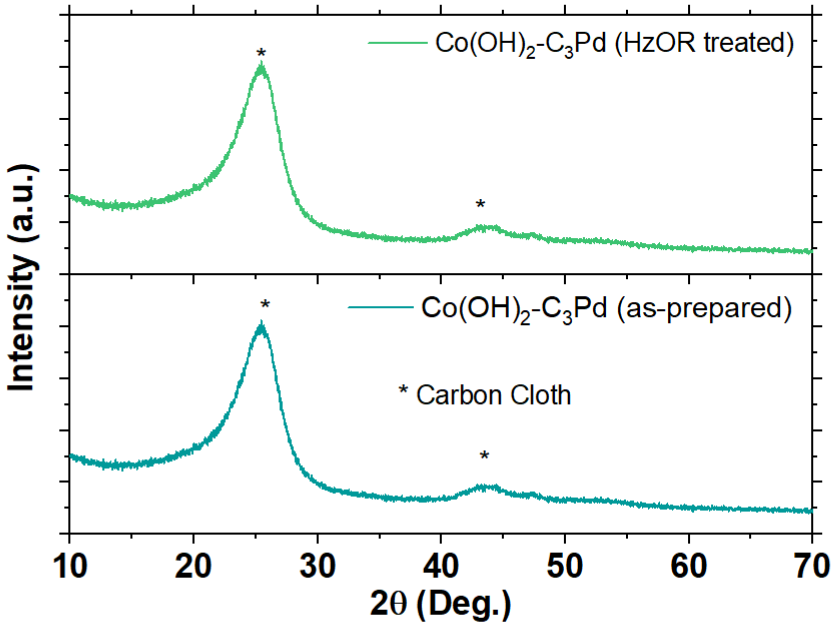

Figure S20. XRD patterns of as-prepared $Co(OH)_2$-C_3Pd and HzOR-treated $Co(OH)_2$-C_3Pd. HzOR treatment condition: 1 LSV at 5 mV s^{-1} executed from -1.1 to -0.70 V vs. Hg/HgO in 1 M KOH + 0.5 M Hz.

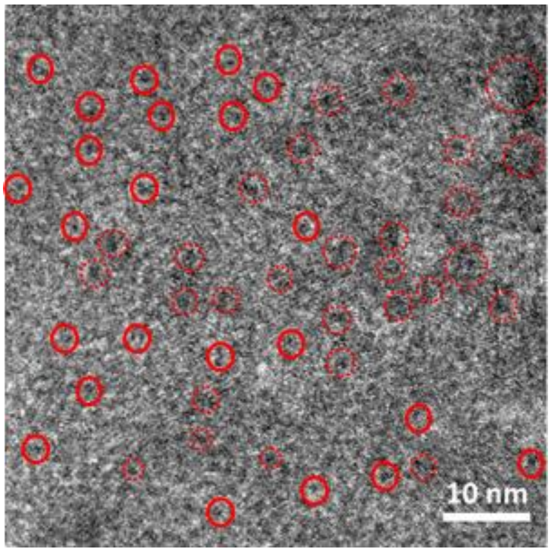

Figure S21. HRTEM image of as-prepared $Co(OH)_2$-C_3Pd showing atomic scale Pd integration with $Co(OH)_2$.

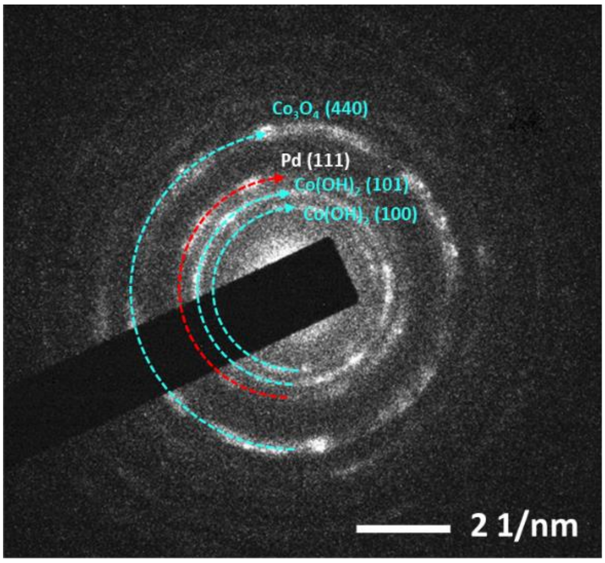

Figure S22. SAD patterns of as-prepared $Co(OH)_2$-C_3Pd.

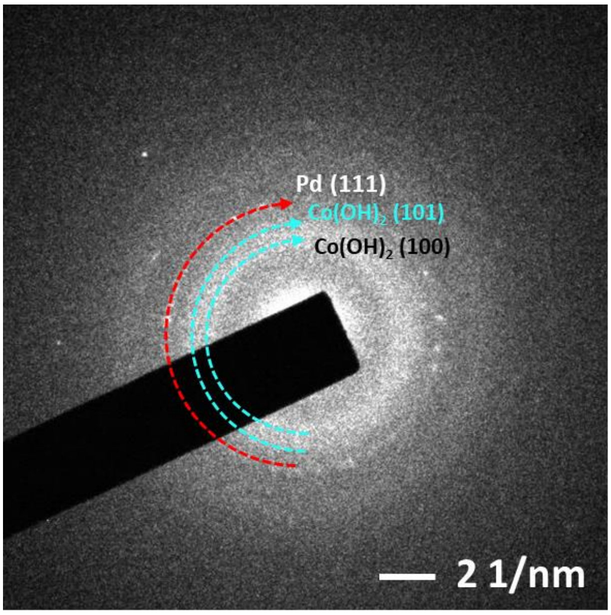

Figure S23. SAD patterns of HzOR treated $Co(OH)_2$-C_3Pd. HzOR treatment condition: 1 LSV executed from -1.1 to -0.70 V vs. Hg/HgO at 5 mV s^{-1} in 1 M KOH + 0.5 M Hz.

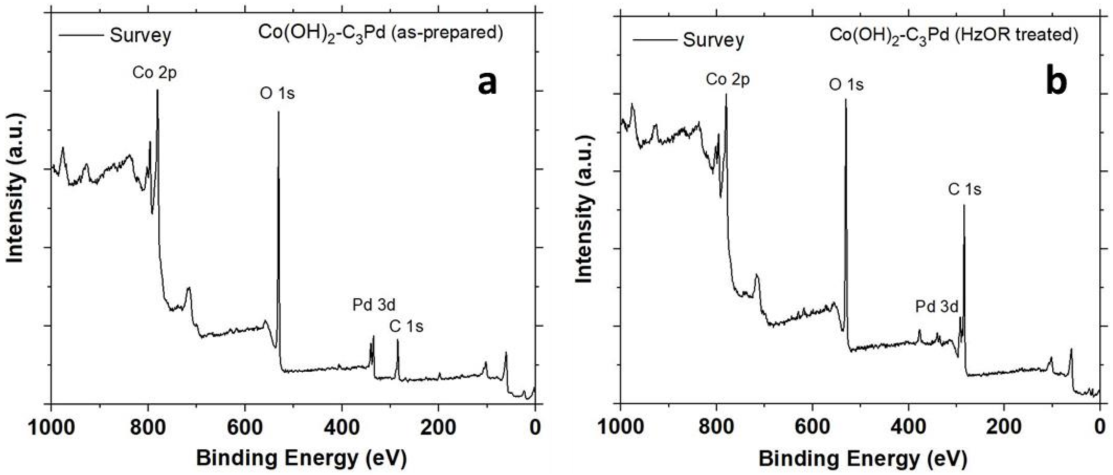

Figure S24. Survey spectra of as-prepared (**a**) & HzOR treated (**b**) $Co(OH)_2$-C_3Pd. HzOR treatment condition: 1 LSV executed from -1.1 to -0.70 V vs. Hg/HgO at 5 mV s^{-1} in 1 M KOH + 0.5 M Hz.

Table S3. Determination of Pd and Co content in different Pd hybrids of $Co(OH)_2$ from ICP-AES analysis.

Samples	Co		Pd	
	**10^{-3} x mg cm^{-2}**	**Atomic % [Bulk Concentration]**	**10^{-3} x mg cm^{-2}**	**Atomic % [Bulk Concentration]**
$Co(OH)_2$-C_1Pd	408.30	~100	~0	~0
$Co(OH)_2$-C_2Pd	421.32	99.427	4.36	0.573
$Co(OH)_2$-C_3Pd	483.05	98.531	12.82	1.469
$Co(OH)_2$-C_4Pd	376.65	95.023	33.85	4.977

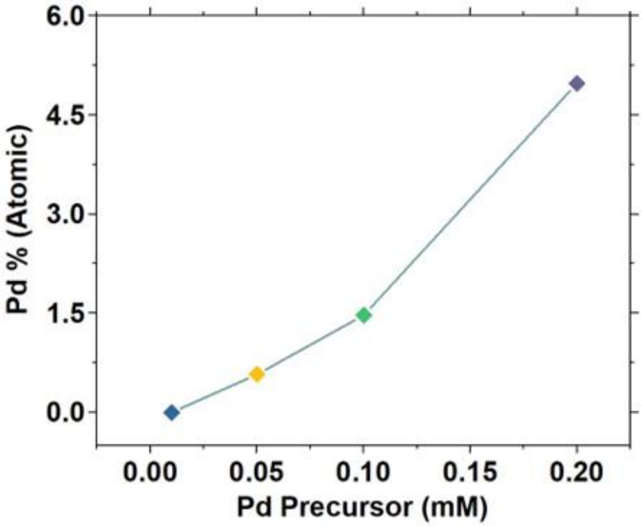

Figure S25. Relationship between Pd content in the catalyst hybrids with $Co(OH)_2$ and corresponding Pd precursor concentration.

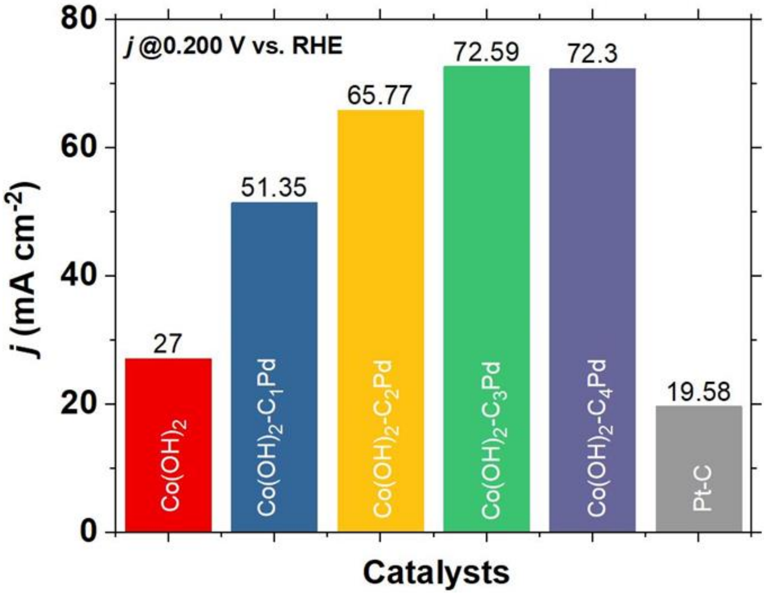

Figure S26. HzOR current densities were calculated from the LSVs at 200 mV for different catalysts generated at 5 mV s^{-1} in 1.0 M KOH + 0.5 M Hz.

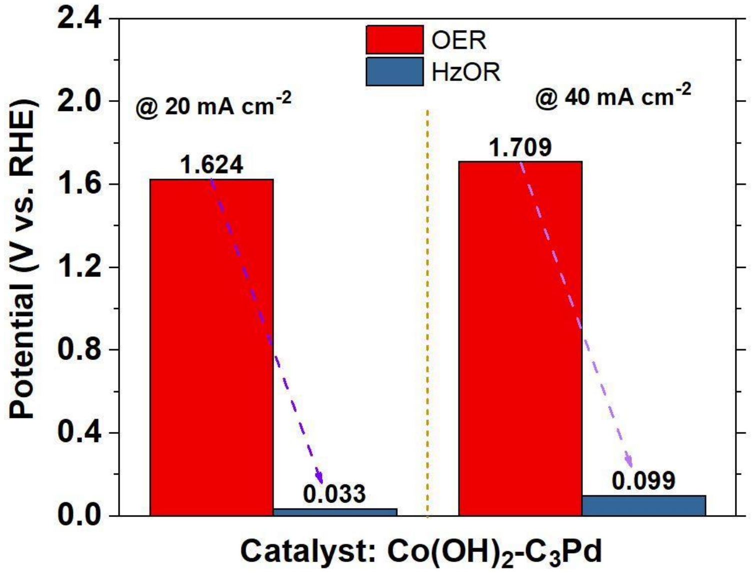

Figure S27. Improvement in the requirement of working potential towards HzOR to generate 20 and 40 mA cm^{-2} compared to OER.

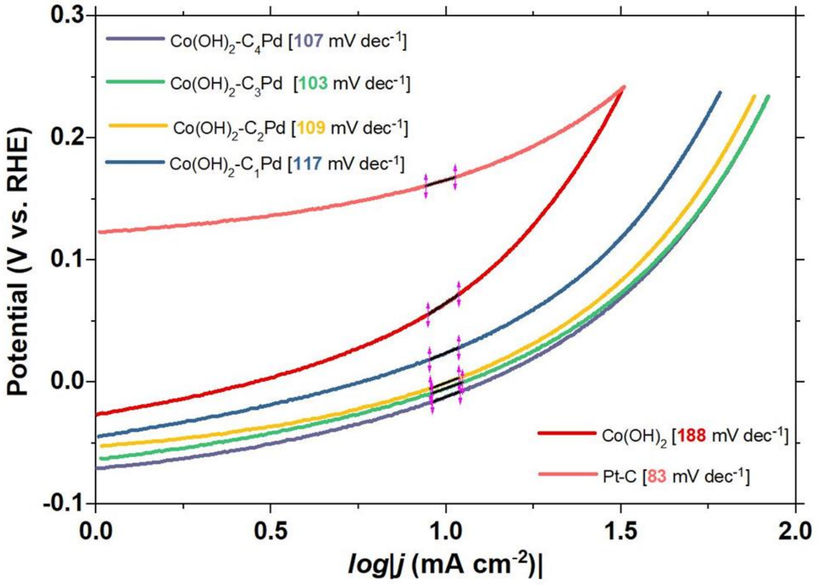

Figure S28. Tafel plots of $Co(OH)_2$, $Co(OH)_2$-C_iPd, and Pt-C were generated from the LSVs.

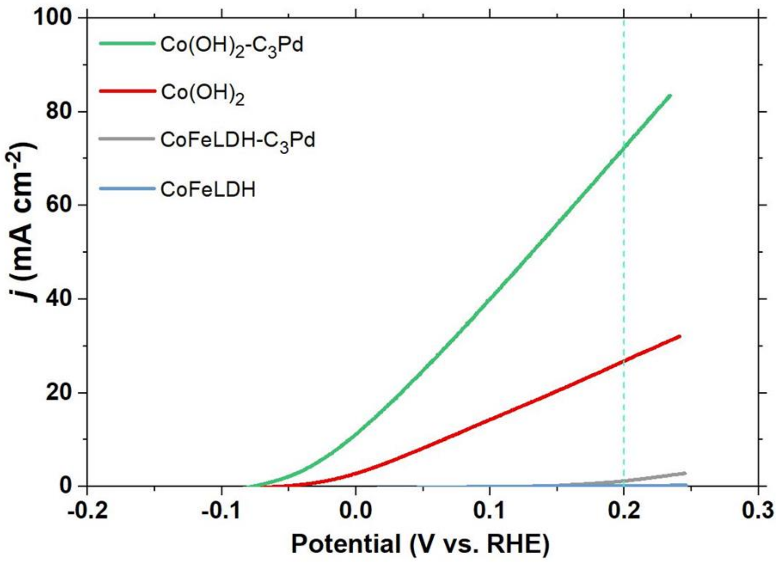

Figure S29. LSV curves of $Co(OH)_2$, CoFeLDH, $Co(OH)_2$-C_3Pd, and CoFeLDH-C_3Pdgenerated at 5 mV s^{-1} in 1.0 M KOH + 0.5 M Hz.

Table S4. Comparison of HzOR activities of various electrocatalysts highlighting the geometric area normalized current densities and mass activities in alkaline (saline) electrolytes with hydrazine.

Catalyst	Electrolyte	Potential at 10 mA cm^{-2}, mV_{10} vs. RHE	Mass Activity		Reference
			i ($A\ mg_M^{-1}$)	Potential (V vs. RHE)	
$Co(OH)_2$-C_3Pd	1 M KOH + 0.5 M Hz	-5	3.48	0.20	This Work
$Co(OH)_2$-C_1Pd		25	11.24	0.20	
$Co(OH)_2$-C_3Pd	1 M KOH + 0.5 M NaCl + 0.5 M Hz	-16	4.40	0.20	This Work
$Co(OH)_2$-C_2Pd		-12	9.83	0.20	
Pd NCs/NiFe	1 M KOH + 0.2 M Hz	50	4.3	0.35	[1]
1% Ce:Co-N-C	1 M KOH + 0.1 M Hz	225	-	-	[2]
RhP_2/Rh@NPG	1 M KOH + 0.1 M Hz	3.1	-	-	[3]
Pd/np-Co_2P	1 M KOH + 0.1 M Hz	~15	-	-	[4]
a-RhPb NFs	1 M KOH + 0.1 M Hz	18	-	-	[5]
l-Rh metalline	1 M KOH + seawater + 0.1 M Hz	-2	-	-	[6]
Mn-CoS_2	1 M KOH + 0.5 M Hz	77	-	-	[7]
Cu_1Ni_2-N	1 M KOH + 0.5 M Hz	0.5	-	-	[8]
V-Ni_3N NS	1 M KOH + 0.1 M Hz	2	-	-	[9]
Ni-C HNSA	1 M KOH + 0.1 M Hz	-20	-	-	[10]
Ir/PNC	1 M KOH + 0.5 M Hz	16.7	~7.5	0.23	[11]
Ni_2P-HNTs/NF	1 M KOH + 0.5 M Hz	18	-	-	[12]
Cu_3P/CF	1 M KOH + 0.5 M Hz	~10	-	-	[13]
Rh/NiFe	1 M KOH + 0.5 M Hz	1380	-	-	[14]
RhIr	1 M KOH + 0.5 M Hz	-12	-	-	[15]
Rh-NS-HCS	1 M KOH + 0.5 M Hz	84	-	-	[16]
Rh/N-CB	1 M KOH + 0.05 M Hz	72	-	-	[17]
Au@Rh ultra-NWs	1 M KOH + 0.1 M Hz	-	~8	0.30	[18]
Ni@Pd/rGO	1 M NaOH + 0.1 M Hz	-	~2.6	0.20	[19]
Rh-SA/$Ti_3C_2O_x$	1 M KOH + 0.1 M Hz	~40	-	-	[20]
ACE-Rh	1 M KOH + 0.5 M Hz	~40	10	0.201	[21]
CC@CoNC-600	1 M KOH + seawater + 0.5 M Hz	-61	-	-	[22]

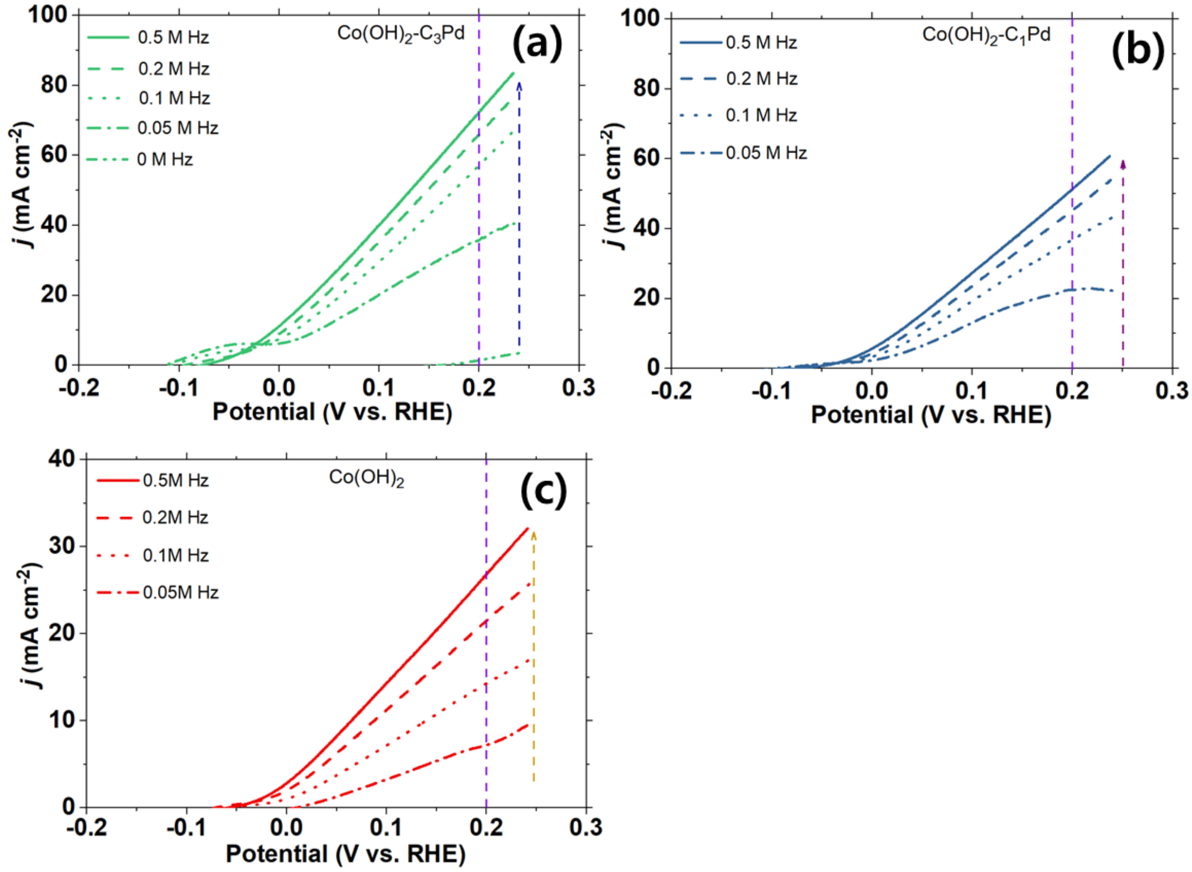

Figure S30. Hydrazine concentration dependent LSV curves of (**a**) $Co(OH)_2$-C_3Pd, (**b**) $Co(OH)_2$-C_1Pd, and (**c**) $Co(OH)_2$ in 1.0 M KOH.

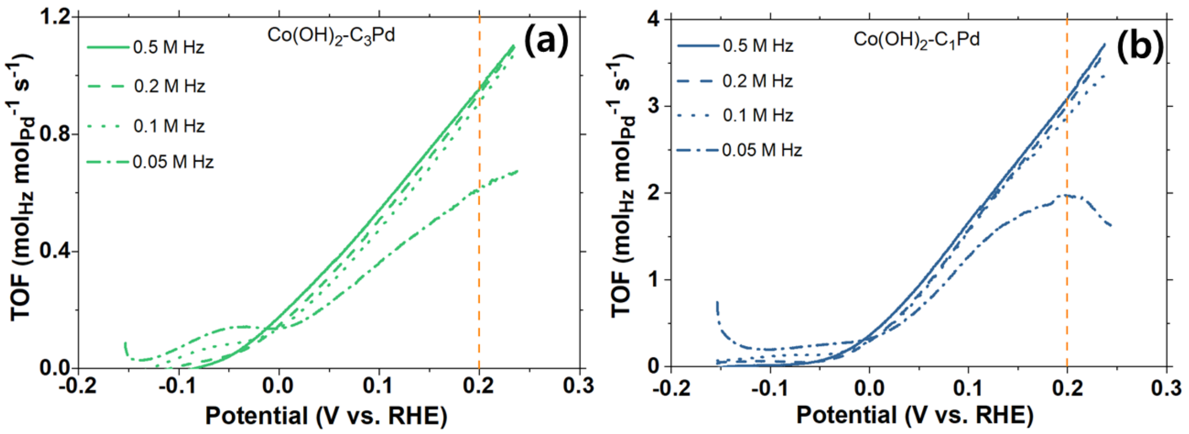

Figure S31. Hydrazine concentration dependent LSV curve derived TOF plots of (**a**) $Co(OH)_2$-C_3Pd and (**b**) $Co(OH)_2$-C_1Pd in 1.0 M KOH.

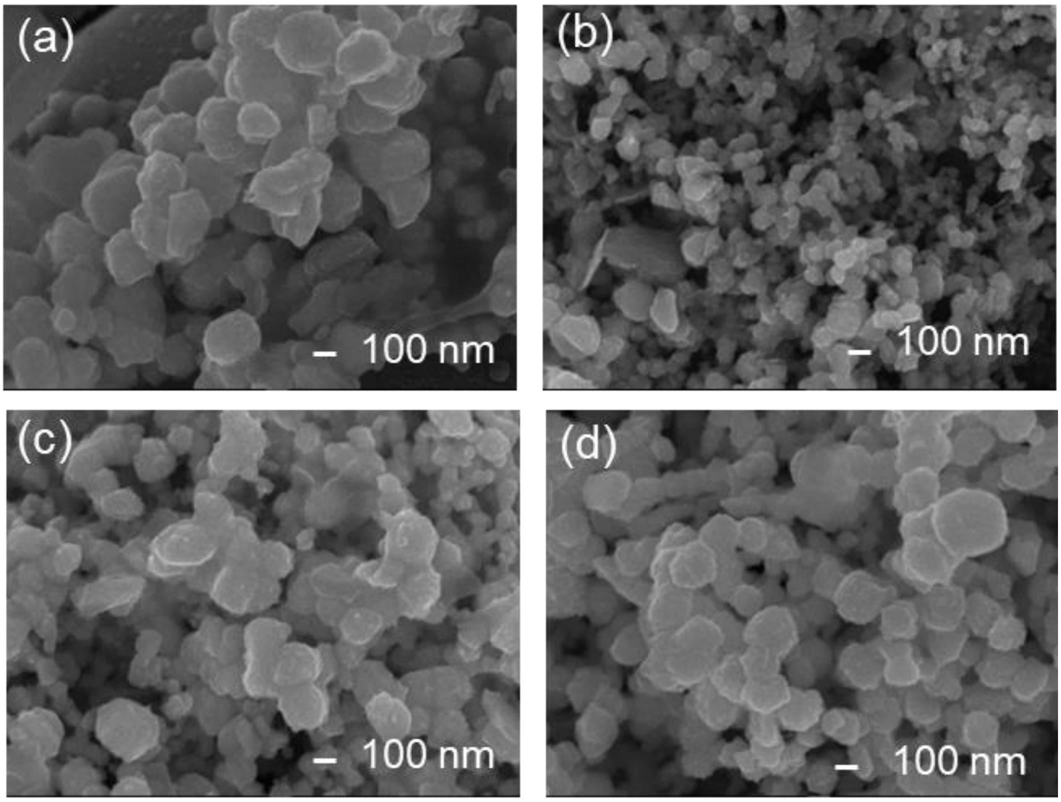

Figure S32. SEM images of $Co(OH)_2$-C_3Pd obtained after different numbers of LSVs in varied hydrazine concentration with 1 M KOH. LSVs were executed from -1.1 to -0.70 V vs. Hg/HgO at 5 mV s^{-1} towards HzOR. Experimental condition: (**a**) 1 LSV HzOR in 1 M KOH + 0.05 M Hz, (**b**)1st LSV HzOR in 1 M KOH + 0.05 M Hz followed by 90 s delay followed by 2nd LSV HzOR in 1 M KOH + 0.1 M Hz, (**c**) 1st LSV HzOR in 1 M KOH + 0.05 M Hz followed by 90 s delay followed by 2nd LSV HzOR in 1 M KOH + 0.1 M Hz followed by 90 s delay followed by 3rd LSV HzOR in 1 M KOH + 0.2 M Hz, and (**d**) 1st LSV HzOR in 1 M KOH + 0.05 M Hz followed by 90 s delay followed by 2nd LSV HzOR in 1 M KOH + 0.1 M Hz followed by 90 s delay followed by 3rd LSV HzOR in 1 M KOH + 0.2 M Hz followed by 90 s delay followed by 4th LSV HzOR in 1 M KOH + 0.5 M Hz. Prior to this test, as-prepared $Co(OH)_2$-C_3Pd was electrochemically treated towards HzOR in 1 M KOH + 0.5 M Hz. Number of LSV: 1.

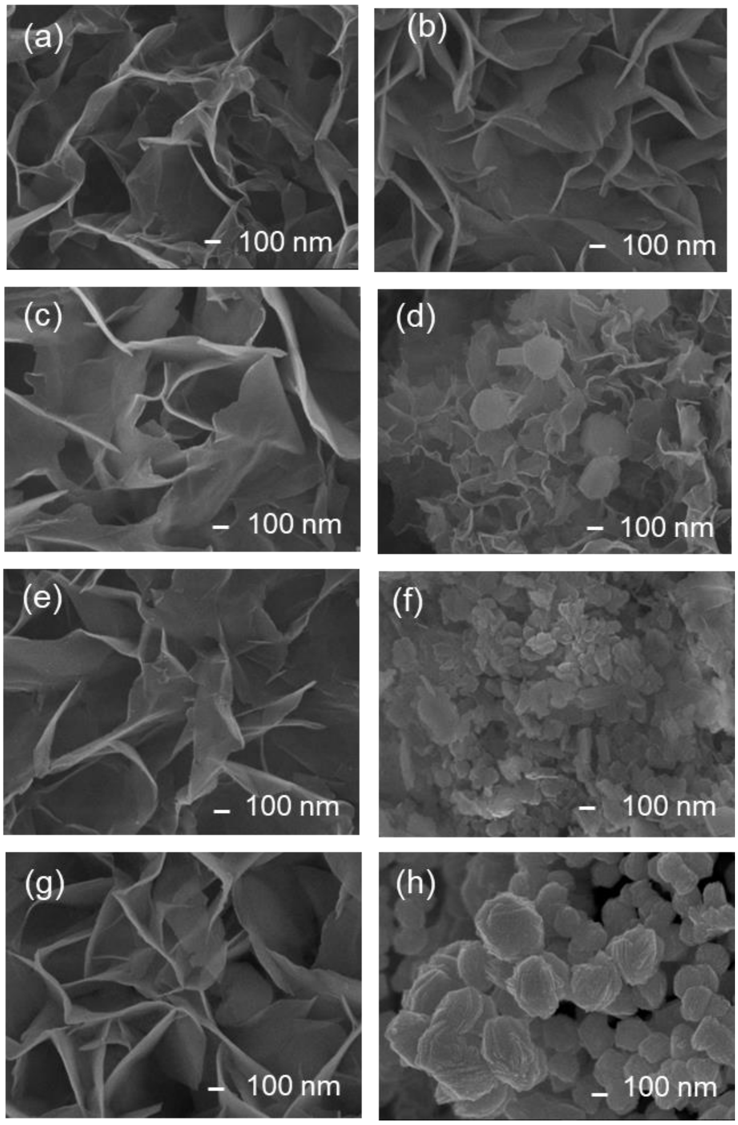

Figure S33. SEM images of $Co(OH)_2$-C_1Pd obtained after different numbers of LSVs in varied hydrazine concentration with1 M KOH. LSVs were executed from -1.1 to -0.70 V vs. Hg/HgO at 5 mV s^{-1} towards HzOR. Experimental condition: (**a, b**) 1 LSV HzOR in 1 M KOH + 0.05 M Hz, (**c, d**) 1st LSV HzOR in 1 M KOH + 0.05 M Hz followed by 90 s delay followed by 2nd LSV HzOR in 1 M KOH + 0.1 M Hz, (**e, f**) 1st LSV HzOR in 1 M KOH + 0.05 M Hz followed by 90 s delay followed by 2nd LSV HzOR in 1 M KOH + 0.1 M Hz followed by 90 s delay followed by 3rd LSV HzOR in 1 M KOH + 0.2 M Hz, and (**g, h**) 1st LSV HzOR in 1 M KOH + 0.05 M Hz followed by 90 s delay followed by 2nd LSV HzOR in 1 M KOH + 0.1 M Hz followed by 90 s delay followed by 3rd LSV HzOR in 1 M KOH + 0.2 M Hz followed by 90 s delay followed by 4th LSV HzOR in 1 M KOH + 0.5 M Hz. Prior to this test, as-prepared $Co(OH)_2$-C_1Pd was electrochemically treated towards HzOR in 1 M KOH + 0.5 M Hz. Number of LSV: 1.

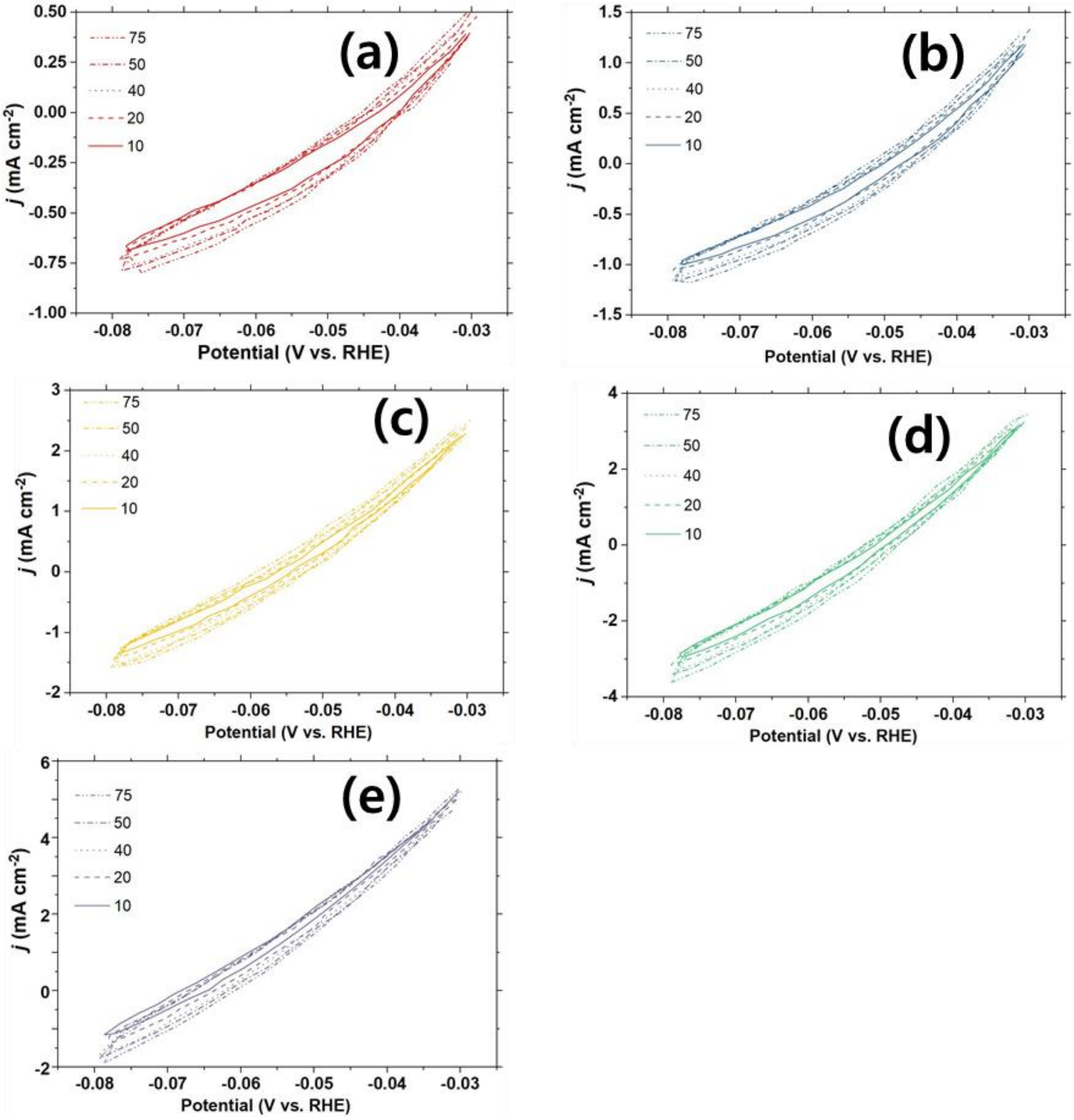

Figure S34. CV curves of (**a**) $Co(OH)_2$ (**b**) $Co(OH)_2$-C_1Pd, (**c**) $Co(OH)_2$-C_2Pd, (**d**) $Co(OH)_2$-C_3Pd and (**e**) $Co(OH)_2$-C_4Pd generated at different scan rates (10, 20, 40, 50, and 75 mV s^{-1}).

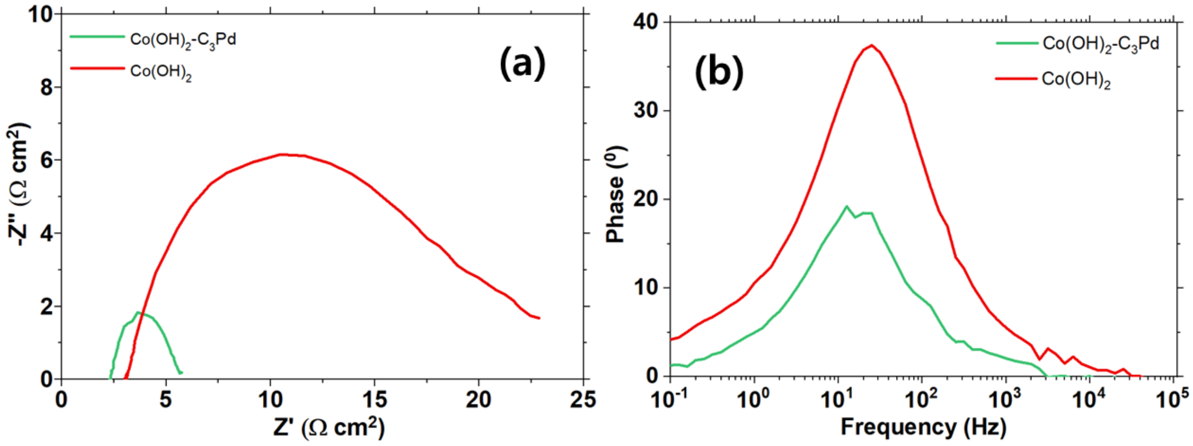

Figure S35. (**a**) EIS and (**b**) Bode phase plots generated in 1 M KOH + 0.5 M Hz.

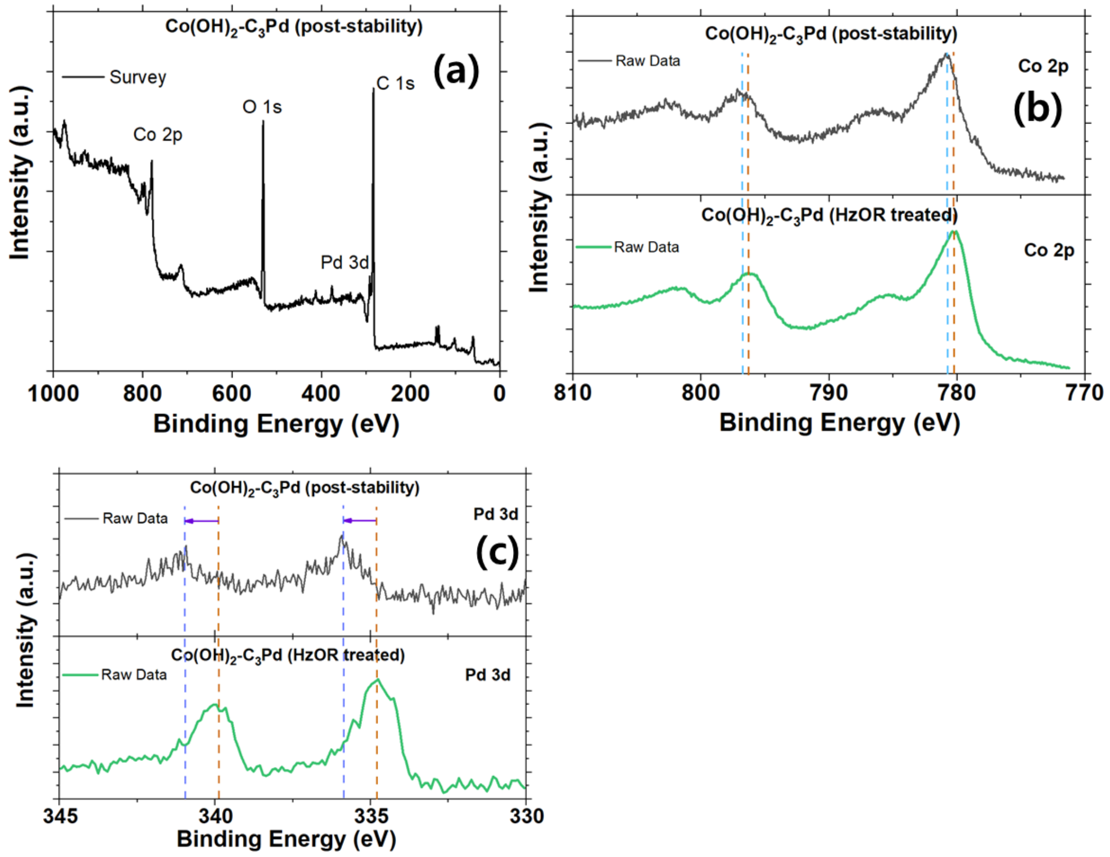

Figure S36. Post-stability (**a**) XPS survey spectra of $Co(OH)_2$-C_3Pd with corresponding (**b**) Co 2p and (**c**) Pd 3d XPS spectra. HzOR electrolyte: 1 M KOH + 0.5 M Hz.

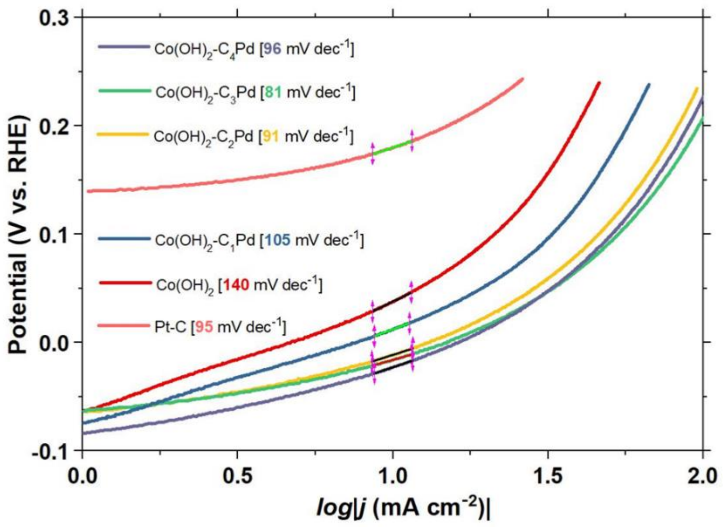

Figure S37. Tafel plots of $Co(OH)_2$, $Co(OH)_2$-C_iPd, and Pt-C generated from the obtained LSVs in 1.0 M KOH + 0.5 M NaCl + 0.5 M Hz.

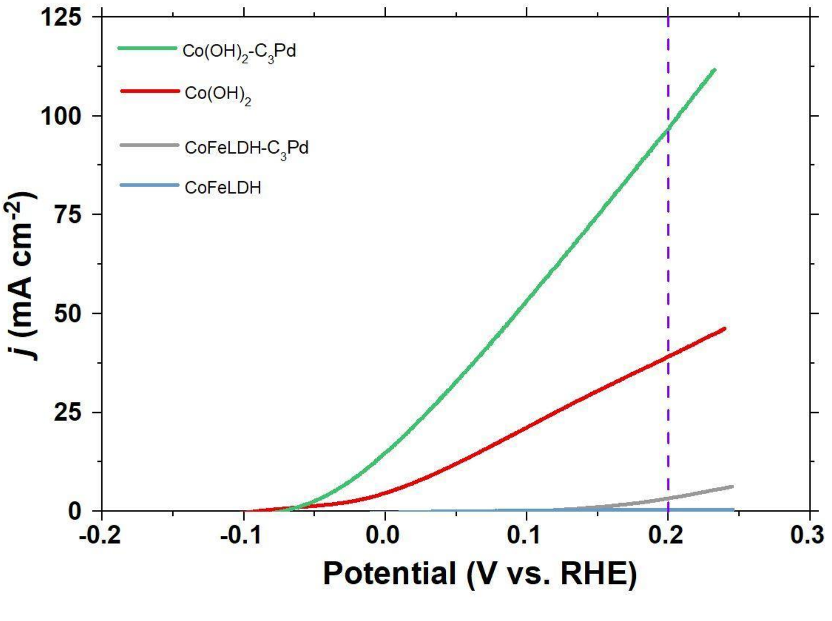

Figure S38. LSV curves of $Co(OH)_2$, CoFeLDH, $Co(OH)_2$-C_3Pd, and CoFeLDH-C_3Pd generated at 5 mV s^{-1} in 1.0 M KOH + 0.5 M NaCl + 0.5 M Hz.

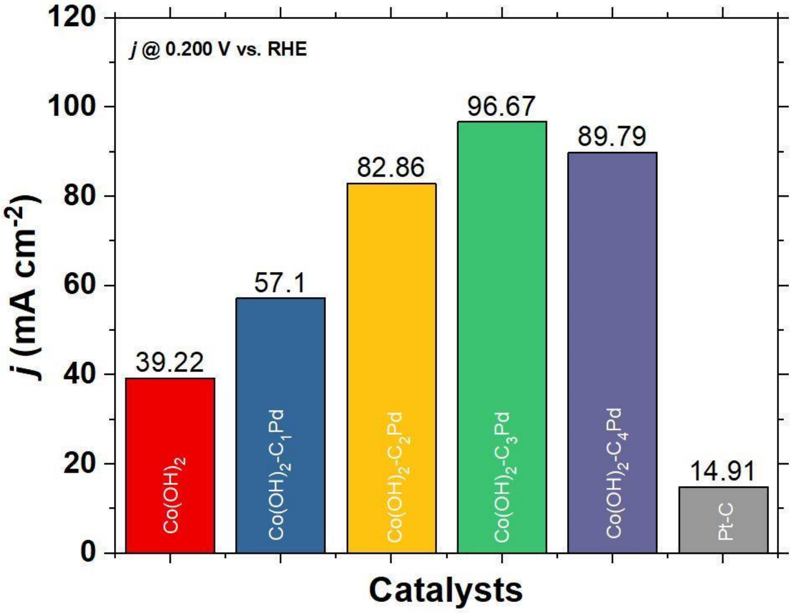

Figure S39. HzOR current densities at 200 mV for different catalysts were obtained in 1.0 M KOH + 0.5 M NaCl + 0.5 M Hz.

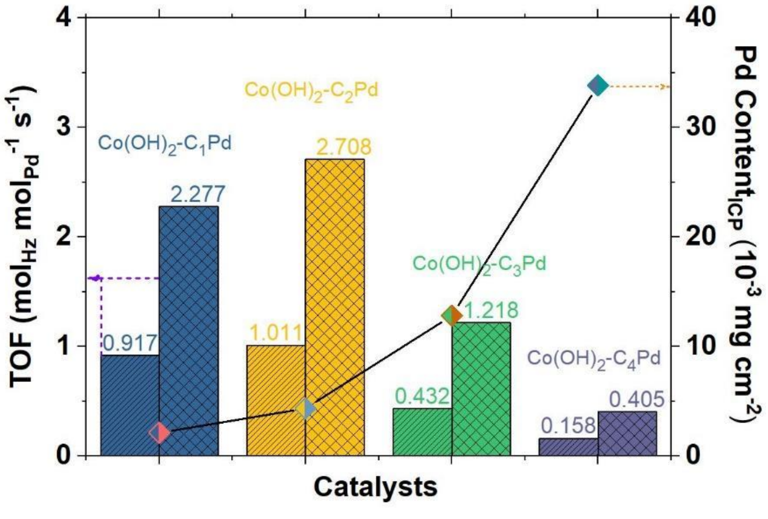

Figure S40. TOF plots generated at 25 mV (with dense line pattern) and 200 mV (with sparse pattern) in conjunction with Pd content obtained from the ICP-AES analysis for $Co(OH)_2$-C_iPd. Electrolyte: 1.0 M KOH + 0.5 M NaCl + 0.5 M Hz.

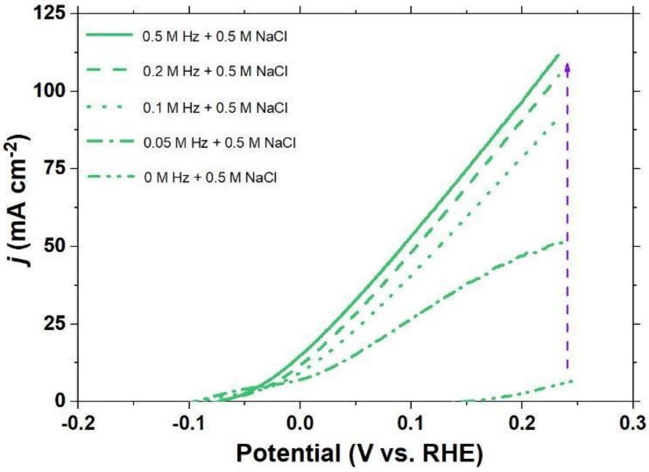

Figure S41. Hz concentration dependent LSVs of $Co(OH)_2$-C_3Pd in 1M KOH + 0.5 M NaCl generated at 5 mV s^{-1}.

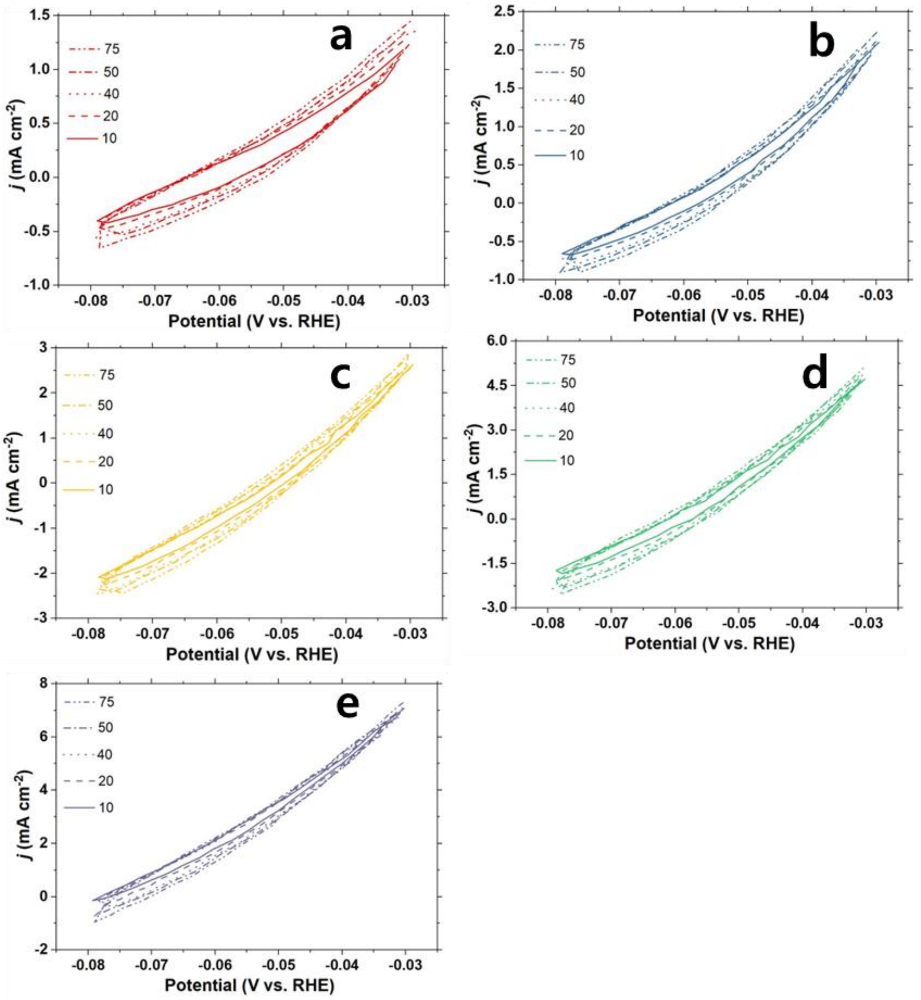

Figure S42. CV curves of $Co(OH)_2$ (a), $Co(OH)_2$-C_1Pd (b), $Co(OH)_2$-C_2Pd (c), $Co(OH)_2$-C_3Pd (d), and $Co(OH)_2$-C_4Pd (e) generate scan rates (10, 20, 40, 50, and 75 mV s^{-1}).

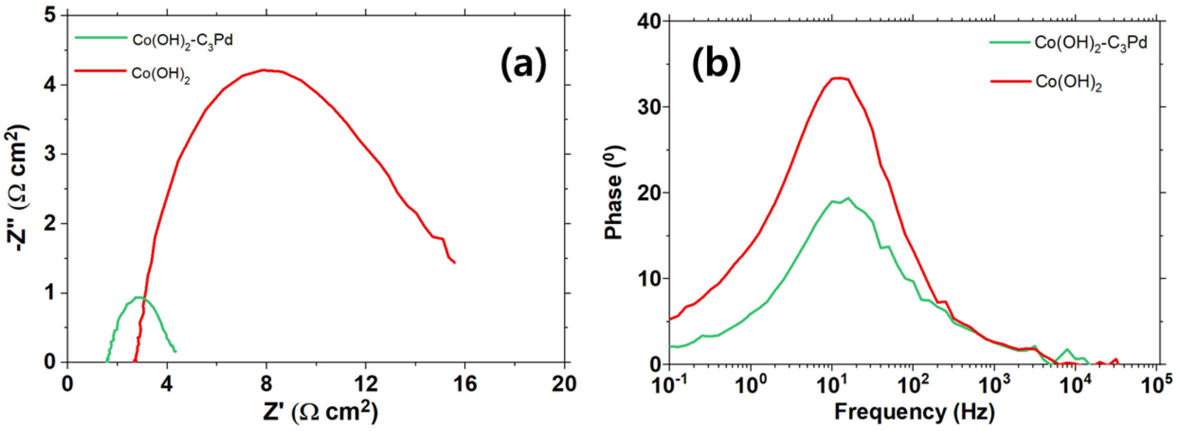

Figure S43. (**a**) EIS and (**b**) Bode phase plots generated in 1 M KOH + 0.5 M NaCl + 0.5 M Hz.

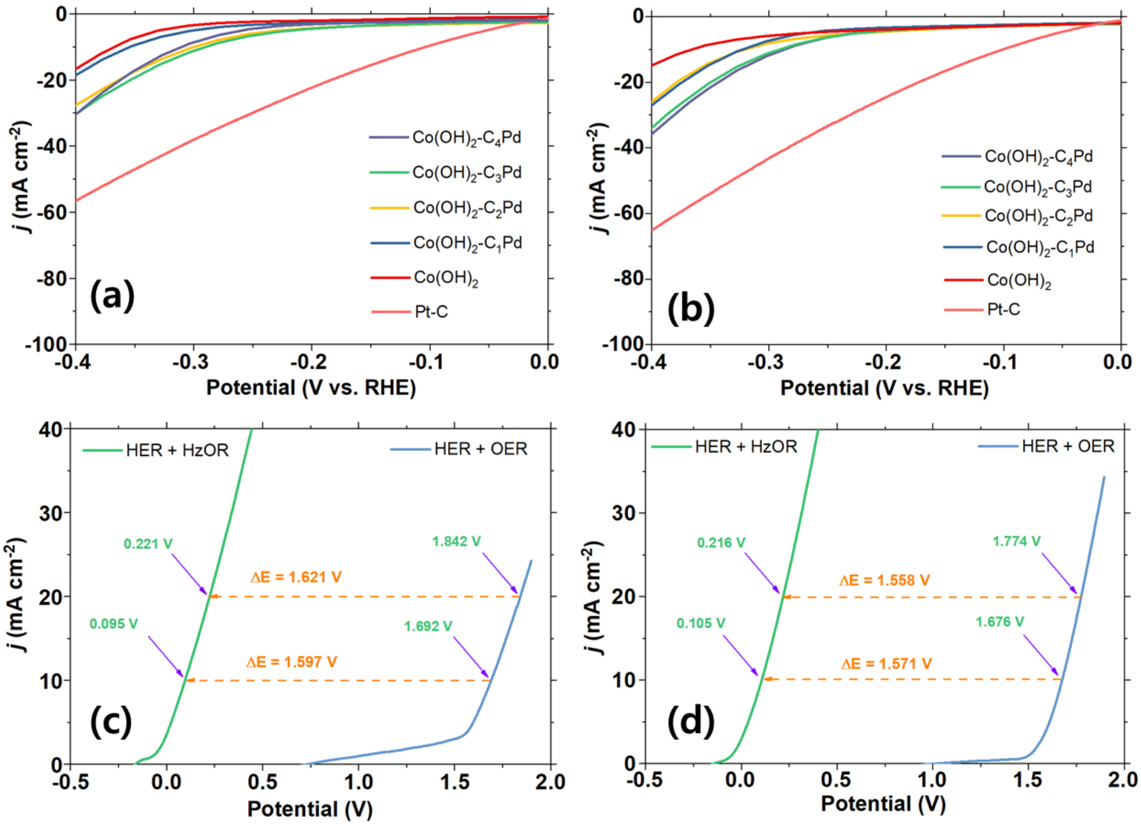

Figure S44. HER activities in (**a**) 1 M KOH and (**b**) 1 M KOH + 0.5 M NaCl. Replacement of OER with HzOR using $Co(OH)_2$-C_3Pd (+) || Pt-C (-) generated in (**c**) 1 M KOH and (**d**) 1 M KOH + 0.5 M NaCl. HzOR was tested for 0.5 M Hz.

Reference

[1] G. Liu, T. Nie, H. Wang, T. Shen, X. Sun, S. Bai, L. Zheng, Y.-F. Song, *ACS Catal.* **2022**, *12*, 10711.
[2] J. Li, C. Dong, M. Guo, W. Gao, L. Kang, F. Lei, P. Hao, J. Xie, B. Tang, *Chem. Commun.* **2022**, *58*, 6845.
[3] Y. Liu, J. Ding, F. Li, X. Su, Q. Zhang, G. Guan, F. Hu, J. Zhang, Q. Wang, Y. Jiang, B. Liu, H. bin Yang, *Adv. Mater.* **2023**, *35*, 2207114.
[4] K. Jiang, Z. Liu, Y.-R. Lu, M. Wang, D. Chen, L. Cai, T.-S. Chan, P. Liu, A. Pan, Y. Tan, *Adv. Mater.* **2022**, *35*, 2207850.
[5] W. Tian, X. Zhang, Z. Wang, L. Cui, M. Li, Y. Xu, X. Li, L. Wang, H. Wang, *Chem. Eng. J.* **2022**, *440*, 135848.
[6] K. Deng, Q. Mao, W. Wang, P. Wang, Z. Wang, Y. Xu, X. Li, H. Wang, L. Wang, *Appl. Catal. B* **2022**, *310*, 121338.
[7] J. Hou, X. Peng, J. Sun, S. Zhang, Q. Liu, X. Wang, J. Luo, X. Liu, *Inorg. Chem. Front.* **2022**, *9*, 3047.
[8] Z. Wang, L. Xu, F. Huang, L. Qu, J. Li, K. A. Owusu, Z. Liu, Z. Lin, B. Xiang, X. Liu, K. Zhao, X. Liao, W. Yang, Y.-B. Cheng, L. Mai, *Adv. Energy Mater.* **2019**, *9*, 1900390.
[9] J. Zhang, Y. Liu, J. Li, X. Jin, Y. Li, Q. Qian, Y. Wang, A. El-Harairy, Z. Li, Y. Zhu, H. Zhang, M. Cheng, S. Zeng, G. Zhang, *ACS Appl. Mater. Interfaces* **2021**, *13*, 3881.
[10] Y. Zhu, J. Zhang, Q. Qian, Y. Li, Z. Li, Y. Liu, C. Xiao, G. Zhang, Y. Xie, *Angew. Chem., Int. Ed.* **2022**, *61*, e202113082.
[11] H.-L. Huang, X. Guan, H. Li, R. Li, R. Li, S. Zeng, S. Tao, Q. Yao, H. Chen, K. Qu, *Chem. Commun.* **2022**, *58*, 2347.
[12] T.-J. Wang, G.-R. Xu, H.-Y. Sun, H. Huang, F.-M. Li, P. Chen, Y. Chen, *Nanoscale* **2020**, *12*, 11526.
[13] M. Liu, R. Zhang, L. Zhang, D. Liu, S. Hao, G. Du, A. M. Asiri, R. Kong, X. Sun, *Inorg. Chem. Front.* **2017**, *4*, 420.
[14] G. Liu, Z. Wang, T. Shen, X. Zheng, Y. Zhao, Y.-F. Song, *Nanoscale* **2021**, *13*, 1869.
[15] M. Zhang, Z. Wang, Z. Duan, S. Wang, Y. Xu, X. Li, L. Wang, H. Wang, *J. Mater. Chem. A* **2021**, *9*, 18323.
[16] D. Qi, S. Liu, H. Chen, S. Lai, Y. Qin, Y. Qiu, S. Dai, S. Zhang, J. Luo, X. Liu, *Mater. Chem. Front.* **2021**, *5*, 3125.
[17] N. Jia, Y. Liu, L. Wang, P. Chen, X. Chen, Z. An, Y. Chen, *ACS Appl. Mater. Interfaces* **2019**, *11*, 35039.
[18] Q. Xue, H. Huang, J. Y. Zhu, Y. Zhao, F. M. Li, P. Chen, Y. Chen, *Appl. Catal. B* **2020**, *278*, 119269.
[19] M. G. Hosseini, R. Mahmoodi, M. Abdolmaleki, *N. J. Chem.* **2018**, *42*, 12222.
[20] X. Peng, Y. Mi, X. Liu, J. Sun, Y. Qiu, S. Zhang, X. Ke, X. Wang, J. Luo, *J. Mater. Chem. A* **2022**, *10*, 6134.
[21] H. H. Lee, D. S. Kim, S. Sarker, J. H. Choi, H. S. Lee, H. K. Cho, *Energy Environ. Mater.* **2022**, *0*, e12556.
[22] Y. Xin, K. Shen, T. Guo, L. Chen, Y. Li, *Small* **2023**, DOI 10.1002/smll.202300019.
